# Cardiorespiratory Fitness and Performance Adaptations to High-Intensity Interval Training: Are There Differences Between Men and Women? A Systematic Review with Meta-Analyses

**DOI:** 10.1007/s40279-023-01914-0

**Published:** 2023-09-07

**Authors:** Merilyn Lock, Ibtisam Yousef, Bridget McFadden, Hend Mansoor, Nathan Townsend

**Affiliations:** 1 Division of Exercise Science, Health and Epidemiology, College of Health and Life Sciences, Hamad Bin Khalifa University, Doha, Qatar; 2 Patient and Family Education Unit, Nursing Department, Hamad Medical Corporation, Doha, Qatar; 3 Department of Exercise Science, Arnold School of Public Health, University of South Carolina, Columbia, SC USA; 4 Department of Family, Nutrition, and Exercise Sciences, Queens College, City University of New York, Queens, NY USA; 5 Department of Pharmacy Practice and Science, College of Pharmacy, University of Kentucky, Lexington, KY USA

## Abstract

**Background:**

It is important to consider biological sex as a variable that might influence exercise adaptation in order to optimize exercise prescription for men and women.

**Objective:**

The aim of this study was to quantify the impact of biological sex on maximal oxygen uptake ($\dot{V}$O_2max_) and performance outcomes after high-intensity interval training (HIIT).

**Methods:**

A systematic search and review was conducted by two independent reviewers up to 8 September 2022 using MEDLINE, SPORTDiscus, and Sports Medicine & Education Index in ProQuest. Trials including healthy adults were included if they presented data for or compared male and female $\dot{V}$O_2max_ or performance outcomes in response to HIIT. Performance outcomes included measures of exercise performance and concurrently measured physiological adaptations. Where appropriate, a random-effects, pre-post meta-analysis was undertaken. Data were sub-grouped for men and women, baseline training level, mean age, intervention type, and intervention length. Heterogeneity was assessed using Chi^2^, Cochran’s *Q*, and Higgins *I*^2^ and sensitivity analyses, where required. Study quality was assessed using the Newcastle–Ottawa Scale and publication bias was assessed through visual inspection of funnel plots.

**Results:**

Thirty-three references from 28 trials were included in the review (*n* = 965; 462 women and 503 men). Meta-analyses included 19 studies for $\dot{V}$O_2max_, eight for peak power output from $\dot{V}$O_2max_ testing (PPO), and five for threshold power (power_AT_). Meta-analyses revealed similar increases in $\dot{V}$O_2max_ in women (*g* = 0.57; 95% CI 0.44–0.69) and men (*g* = 0.57; 95% CI 0.42–0.72), and power_AT_ in women (*g* = 0.38; 95% CI 0.13–0.64) and men (*g* = 0.38; 95% CI 0.11–0.64). Raw mean differences for change in $\dot{V}$O_2max_ were Δ 0.32 L·min^−1^ and 3.50 mL·kg^−1^·min^−1^ in men, versus Δ 0.20 L·min^−1^ and 3.34 mL·kg^−1^·min^−1^ for women. No significant sex differences were present for the primary analysis of any outcome. After sub-grouping, significant differences were present for PPO where the effect size was higher for well-trained women (*g* = 0.37) compared with well-trained men (*g* = 0.17), and for $\dot{V}$O_2max_ where interventions with a duration of 4 weeks or less had significantly smaller effect sizes compared with those longer than 4 weeks (*p* < 0.001). Unweighted mean percentage change in $\dot{V}$O_2max_, PPO, and power_AT_ across studies was 11.16 ± 7.39%, 11.16 ± 5.99%, and 8.07 ± 6.55% for women, and 10.90 ± 5.75%, 8.22 ± 5.09%, and 7.09 ± 7.17% for men, respectively. Significant heterogeneity was present for both $\dot{V}$O_2max_ and PPO (*I*^2^, range: 62.06–78.80%). Sub-grouping by baseline training status and intervention length decreased heterogeneity in most groups. A qualitative synthesis of other outcomes indicated similar improvements in fitness and performance for men and women with some evidence suggesting differences in the mechanisms of adaptation.

**Limitations and Risk of Bias:**

Publication bias is unlikely to have significantly influenced results for $\dot{V}$O_2max_ or power_AT_, but the meta-analysis of PPO could have benefitted from additional study data to strengthen results. The overlap in age categories and sensitivity of the analysis limits the accuracy of the results of the sub-grouping by age.

**Conclusions:**

Findings indicated no sex-specific differences for any fitness or performance outcomes. Baseline training status and intervention length accounted for most variability in outcomes. PROSPERO registration number: CRD42021272615.

**Supplementary Information:**

The online version contains supplementary material available at 10.1007/s40279-023-01914-0.

## Introduction

High-intensity interval training (HIIT) involves repeated bouts of exercise performed in the vigorous intensity domain interspersed with periods of complete rest or low-intensity exercise. High-intensity intervals can last anywhere from 5 s to 8 min and are generally performed above the second ventilatory threshold which elicits a heart rate response between 80 to 100% of heart rate maximum [1]. Variables such as the interval duration, intensity, and the number of work and recovery bouts can be manipulated to develop a myriad of different HIIT protocols. When the work rate increases towards the upper bound of the typical intensity range for HIIT, a specific form of HIIT referred to as ‘sprint interval training’ (SIT) occurs. This involves very brief work bouts, usually around 8–30 s, that are repeated and performed at supra-maximal intensity (greater than that associated with 100% of $\dot{V}$O_2max_) or ‘all-out’ efforts [2]. These relatively short intervals are also interspersed with either long or short recovery periods [3].

Previous studies have shown that HIIT can elicit equal or greater improvements in maximal oxygen uptake ($\dot{V}$O_2max_) compared with continuous training [4–7], particularly with the use of higher intensities and longer work intervals [8, 9]. Currently, HIIT is increasingly prescribed as a potential therapeutic intervention to address a variety of chronic illnesses including cardiovascular disease, cancer, and metabolic syndrome, due to the robust evidence showing significantly enhanced cardiorespiratory fitness [6, 10–13]. The approach appears to be a viable strategy for fostering mental, psychological, and cognitive health and may reduce the severity of anxiety and depression [14–19]. Lastly, HIIT can be undertaken without the need for expensive gym equipment or access to commercial exercise training facilities. Overall, HIIT appears to be a feasible alternative to traditional endurance training for improving cardiorespiratory fitness, and may facilitate these changes with a surprisingly low training volume [20, 21].

Anatomical and physiological differences between men and women are believed to underlie differences in $\dot{V}$O_2max_ and endurance performance [22]; however, differences in the adaptation to chronic training are less well known. Studies in which biological sex was treated as an independent variable have been considered crucial towards improving the understanding of overall human health, and also for enabling more personalized, sex-specific training regimens [23]. Compared with men, the absolute aerobic capacity of trained women is 10–25% lower, however, when maximal oxygen uptake ($\dot{V}$O_2max_) is adjusted relative to body weight, the difference can be reduced to around 5–10% [24–26]. After normalization of body weight, the remaining difference could be due to lower blood hemoglobin concentration, cardiac dimensions, and total blood volume [24, 27–29]. For example, women's hearts and major blood vessels are typically smaller than those of men of the same body weight, ethnicity, and chronological age [22, 30–33]. Similarly, various studies have identified respiratory system limitations in women. Compared with men, height- and weight-matched women appear to have smaller lung sizes [34–36]. Furthermore, the diameter of the conducting airways is lower and the number of alveoli is less than in men, both of which negatively affect airflow and efficiency of gas exchange during heavy exercise [36–39]. Although some research indicates that performance could be impacted by sex differences in lung volume, but not airway anatomy and mechanics [22, 40, 41], such differences likely still contribute to physiological limitations to oxygen transport and thus would tend to exert a negative impact on exercise performance in women compared with age-, height- and/or weight-matched men.

Another physiological sex difference that has the potential to influence exercise response is that the less fatigable type I muscle fibers tend to be more abundant in women [42]. As such, there is evidence that for the same period of high-intensity exercise, women tend to experience less peripheral muscle fatigue-related contractile dysfunction than men, which translates to greater fatigue resistance and faster recovery [43]. Due to the differences in muscle fiber type percentages, women oxidize more fat and less protein and carbohydrate at matched relative intensity compared with men [44], whereas men possess higher glycolytic capacity [45, 46], which would therefore tend to alter intracellular homeostasis to a greater extent in men versus women at an equivalent relative intensity. Moreover, in response to HIIT or SIT, some studies have reported that females present with lower blood lactate levels [47]. Other findings have demonstrated that anaerobic capacity, estimated by energetic equivalents of the phosphagen and glycolytic pathways, may be lower in women when compared with men after a supramaximal effort [48]. Collectively, these studies suggest that women are less prone to peripheral muscle fatigue and have a greater tendency towards more aerobic metabolism than men.

To date, many reviews investigating the role of sex differences on acute exercise responses and chronic adaptation have been narrative in nature [2, 49]. One review concluded that attenuated blood lactate accumulation, lower protein synthesis, and mitochondrial biogenesis occur in women relative to men following SIT [2]. One systematic review, which also included a meta-regression [50], examined the effects of low-volume HIIT on cardiorespiratory fitness in adults and found moderate improvements in the $\dot{V}$O_2max_ of active and sedentary participants, without presenting a conclusion regarding a sex-specific response to HIIT. A more recent meta-analysis concluded that HIIT is an efficient method of decreasing total abdominal and visceral fat mass without differences between men and women, but it did not investigate cardiorespiratory fitness as an outcome [51]. A meta-analysis by Diaz‑Canestro and Montero [52] found significantly larger increases in both absolute and relative $\dot{V}$O_2max_ after moderate-intensity endurance training in men compared with women; however, this review did not investigate the effects of HIIT as an intervention. Overall, the findings of these reviews indicate the potential for sex to impact health outcomes and cardiorespiratory fitness adaptations to exercise training, yet to date, no definitive conclusions can be drawn regarding how sex differences influence the adaptation to HIIT. Therefore, the objective of this systematic review with meta-analyses was to examine the influence of biological sex on the relative magnitude of adaptations in cardiorespiratory fitness and performance, following either HIIT or SIT interventions.

## Methods

### Development of the Research Question

To address the objective of the review, the research question was formulated using the Population, Intervention, Comparison, Outcome (PICO) framework as follows:

Is the relative magnitude of adaptation of maximal cardiorespiratory fitness and measures of performance (*outcome*) in response to HIIT or SIT (*intervention*) in healthy adults (*population*) different between men and women (*comparison*)?

### Literature Search and Screening

This systematic review has been registered on the PROSPERO International Prospective Register of Systematic Reviews (registration number: CRD42021272615). Additionally, this review has been conducted and reported according to the Preferred Reporting Items for Systematic Reviews and Meta-analyses (PRISMA) guidelines [53].

A search of three major electronic databases (MEDLINE, Sports Medicine & Education Index in ProQuest, and SPORTDiscus) was undertaken through to 8 September 2022. The keywords used during the search were ‘high intensity’, ‘high-intensity’, ‘HIIT’, ‘HIT’, ‘intervals’, ‘intermittent’, ‘sprint’, ‘HIIE’, ‘vigorous’, ‘maximal’, ‘exercise’, ‘workout’, ‘intervention’, ‘physical activity, ‘activity’, ‘training’, ‘gender’, ‘sex’, ‘male’, ‘males’ ‘man’, or ‘men’ and ‘female’, ‘females’ ‘woman’, or ‘women’. Subject (MeSH) headings were used for ‘exercise’, ‘exercise training’, ‘exercise adaptation’, and ‘physical activity’ in MEDLINE and SPORTDiscus. The search strategy was recreated in the Sports Medicine & Education Index in ProQuest without the option of subject headings. The ProQuest search was set to search ‘everything except full text’, including title, abstract, and keywords. The full search strategy as it was undertaken in MEDLINE is outlined in Supplementary Online Resource 2 (see electronic supplementary material [ESM]). In addition, the reference lists of previous reviews relevant to HIIT were manually screened to identify any relevant references that were not included in the electronic search. All references captured in the search and identified from reference lists were exported into Zotero reference management software (version 5.0.96.2, USA), and subsequently imported into Covidence online review management program (Australia) for the study selection phase of the review.

Title, abstract screening, and full-text screening were conducted through the Covidence website by two independent reviewers (IY and ML). Any conflicts during the screening process were resolved via consultation between the two reviewers to confirm the reasons underlying inclusion or exclusion. A third reviewer was available for any conflicts that could not be resolved between the first two reviewers.

For inclusion in the review, studies were required to have implemented a HIIT or SIT protocol intervention in a cohort of adults including male and female participants; to have measured cardiorespiratory fitness or performance outcomes; to have presented separate outcomes for men and women, or individual data including sex, and/or presented results of a sex × HIIT analysis for the outcomes of interest. This approach to study inclusion was taken in order to control for confounding from different HIIT/SIT protocols across studies, and to ensure that exercise dose is normalized between male and female sub-groups. Peer-reviewed publications or unpublished theses that were written in English were included in the review. Participant groups were excluded from the review if clear pathology was present (i.e., if diagnosed diseases or disorders were a focus of the intervention) or if the intervention included major confounding factors (i.e., dietary supplementation or manipulation, pharmaceutical or herbal intervention, bed rest, or if HIIT/SIT was not the primary cardiorespiratory exercise intervention). Studies and participant groups with risk factors for disease were included if diagnosed disease states were not present. Research designs including reviews, previous meta-analyses, conference abstracts, case studies, and non-scientific articles were excluded from the review. Outcome measures included any measures relating to cardiorespiratory fitness, maximal or sub-maximal exercise performance including power, anaerobic threshold, or speed-related measures (i.e., time trials and sprints). Musculoskeletal performance outcomes such as field tests of muscular power, strength, or endurance were outside of the scope of the current review. No limitations were placed on the type of measure used for fitness or performance outcomes (i.e., measured vs estimated $\dot{V}$O_2max_, or lab-based vs field tests of performance) or whether or not the study achieved a positive effect overall.

### Assessing the Risk of Bias Within Studies

The risk of bias for each of the individual included trials was evaluated independently by two authors (IY and ML) using the Newcastle–Ottawa Scale (NOS) for quality assessment of case–control studies [54]. To address the unique research question of the current review, the research team needed to focus on an observational element (biological sex differences in outcomes) within interventional studies, therefore a tool for assessing the risk of bias in observational studies was deemed to be more appropriate than a tool to assess the risk of bias within experimental studies. This approach has been previously used for another meta-analysis with a similar research question [52]. Additionally, to address the risk of bias specific to the research question, the comparison of men and women was applied to the NOS in place of cases versus controls.

Due to the risk of low inter-rater reliability associated with the subjective interpretation of the NOS and the previously highlighted need for more detailed guidance around the application of the scale [54–56], additional directions were developed by the research team to apply the NOS to the specific objectives of the current review (see Supplementary Online Resource 3 in the ESM). Studies were scored on a scale of nine in accordance with the NOS scoring system. Any conflicts in the quality rating scores of individual items within each study were resolved through discussion between the researchers. Inter-rater reliability for individual items of the NOS was calculated as the number of trials with the same score from both reviewers before conflict resolution as a proportion of the total number of trials. Domain scores were used to categorize studies into *good*, *fair*, and *poor* quality using the thresholds outlined by the Agency for Healthcare Research and Quality (AHRQ) [57].

### Data Extraction and Meta-Analysis

Data including reference identification information, details of the participant characteristics such as age and target population, details of the intervention (intervention length, HIIT protocol, frequency, intensity, and exercise mode), methods of fitness or performance outcome testing, and results were extracted using a customized Microsoft Excel spreadsheet and Microsoft Word tables. Additionally, any concurrently measured, potentially influential physiological variables such as those relating to cardiac, muscular, or cellular metabolic adaptations, and measures of blood lactate accumulation or lactate clearance were also extracted. In cases where raw data necessary for meta-analysis was not directly reported but could be determined from the available information, it was calculated according to Cochrane recommendations [58]. Similarly, where individual trials included multiple intervention groups that met inclusion criteria, the groups were pooled according to Cochrane recommendations [58]. In cases where outcomes were only presented in figure format, the necessary data was extracted using WebPlotDigitizer software (version 4.5, Ankit Rohatgi, United States of America).

Where possible, pre-and post-intervention outcome data were meta-analyzed using the Meta-Essentials package (Erasmus University, the Netherlands) for Microsoft Excel [59] using differences for dependent groups and continuous data [60]. The dependent measures meta-analysis used a random-effects model and was based on Hedges’ *g*. The magnitude of the effect was inferred based on the exercise science-specific thresholds of small (0.20), moderate (0.60), and large (1.20) standardized mean differences, as outlined by Hopkins and colleagues [61]. Correlation coefficients for individual studies were calculated using mean outcome and change data by applying Follmann’s equation [62]. A mean of the calculated correlation coefficients from studies that provided the necessary data was used to impute a correlation coefficient for all other studies. Standardized mean differences were used to account for variation in the units of measure that were presented across different studies, however, wherever possible the most frequently reported unit of measure for each given outcome was included in the meta-analysis to maximize consistency. In cases where the first and second anaerobic thresholds were reported, the second threshold was used for inclusion in the meta-analysis.

### Sensitivity Analyses and Methods for Exploring Heterogeneity

The primary research question was addressed by sub-grouping male and female data. Sensitivity analysis was then undertaken to check the effect of study quality and any observed outlying studies on total effect size and heterogeneity within the meta-analysis. Outlying studies were removed from the analysis if they were observed to have a poor fit with the remaining studies and an underlying methodological explanation for this was identified. Studies that were categorized as poor on the NOS were excluded for sensitivity analysis to assess the effect of study quality and subsequently excluded from all meta-analyses if the effect was deemed significant. An additional sensitivity analysis was undertaken on the primary analysis of $\dot{V}$O_2max_ to check the effects of the meta-analytical approach and to estimate raw mean differences for the pooled data. This analysis was undertaken in order to provide more practical estimates of baseline values and effects, as well as checking whether the use of raw mean differences over standardized mean differences would result in any changes in the overall findings. Data were pooled in Review Manager (RevMan) version 5.4.1 (*The Cochrane Collaboration*) using a random effects model and a raw mean difference directly comparing baseline absolute and relative $\dot{V}$O_2max_ for men and women, as well as pre-post measures for $\dot{V}$O_2max_ (absolute and relative).

Potential sources of heterogeneity were explored through additional sub-grouping by the pre-determined population characteristics of baseline training status and mean group age, as well as by intervention type and length. Baseline training status was categorized as *untrained*, *moderately trained*, and *well trained* based on the population description by the authors of the primary studies. The grouping of studies into each training status category was confirmed by cross-checking the category against the mean baseline $\dot{V}$O_2max_, where available. In cases where the description of baseline training status was not clear within the primary study, grouping was informed by the baseline $\dot{V}$O_2max_ and the homogeneity with other studies in the grouping. Untrained populations were identified as previously sedentary or those not currently participating in regular exercise at baseline. Moderately trained populations included recreationally active individuals. Well-trained populations included those described as ‘well trained’, and elite, or semi-elite athletes. Sub-grouping by age was undertaken using the mean sample group age and categorized as (a) adults under 30 years; (b) participants aged 30–45 years, and (c) participants over 45 years of age. Sub-grouping into intervention type involved categorizing study data into HIIT (interventions using sub-maximal intensities) or SIT (supra-maximal/all-out intensities). Sub-grouping by intervention length involved categorizing study data into interventions ≤ 4 weeks, 5–9 weeks, and ≥ 10 weeks in duration.

### Qualitative Synthesis

For outcomes where only a small number of trials reported data, and meta-analytical methods were deemed to be inappropriate, results were synthesized qualitatively by grouping various measures and reporting the relevant results of each study.

### Assessing the Risk of Bias Across Studies

The risk of bias across studies was assessed using visual inspection of the funnel plots for the primary meta-analyses [63, 64]. The presence of publication bias was assumed if notable asymmetry was present within the funnel plot. Adjusted effect sizes were reported where relevant.

## Results

### Search Results and Study Characteristics

**Fig. 1:**
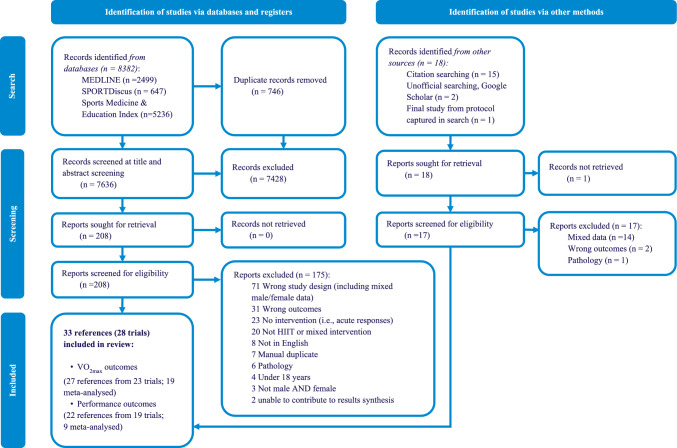
Preferred Reporting Items for Systematic Reviews and Meta-Analyses (PRISMA) flow chart of the search and screening process. *HIIT* high-intensity interval training, $\dot{V}$*O*_*2max*_ maximal oxygen uptake

A total of 33 references from 28 individual trials including 965 participants (462 women and 503 men) were included in the review. One study was initially included in the review but was subsequently excluded due to the inclusion of only two female participants, resulting in the inability to compute an effect size in the meta-analysis [65]. A sex × HIIT analysis was not undertaken by the study authors for the same reason and therefore despite meeting all inclusion criteria the reference could not contribute to the results of the review. The flow of references through the search and screening process is shown in the PRISMA diagram in Fig. 1. The study and population characteristics are shown in Table 1. A summary of the training protocols used is shown in Table 2. Results of the primary meta-analyses for all outcomes and the sub-grouping by participant characteristics are shown in Table 3. Results of sub-groupings by intervention characteristics are shown in Table 4. Individual study results for all fitness and performance outcomes and measures of physiological adaptation are shown in Tables 5, 6, 7 and 8. The PRISMA checklist for the reporting of review methods and results can be found in Supplementary Online Resource 4 in the ESM.

**Table 1:** Study and population characteristics for included studies

Study	Study type	Group	Females		Males		Combined		Population	NOS	
			*n*	Age, y (± SD)	*n*	Age, y (± SD)	*n*	Age, y (± SD)		(Score/9)	Categorization AHRQ
Astorino 2011 [76] + Astorino 2012 [66]	NRCT	SIT	9	25.2 (3.1)	11	25.3 (5.5)			Recreationally active men and women	7	Good
		CON	4	22.6 (3.1)	5	23.0 (2.7)					
Bagley 2016 [77] + Bagley 2021 [78]	NCT	SIT	17	41 (SEM 3.2)	24	38 (SEM 2.7)			Healthy adults (unhealthy subjects, or those involved in competitive sports or cycled for > 15 min/d for ≥ 3 d/wk were excluded)	6	Fair
Bornath 2022 [79]	NCT	HIIT	15	23 (1)	18	23 (2)			Recreationally active adults	7	Good
Bostad 2021 [80]	NCT	SIT	9		6		15	21 (2)	Healthy untrained adults	7	Good
Cicioni-Kolsky 2013 [98]	RCT	HIIT	9	18.4 (1.3)	10	20.8 (3.8)			Recreationally and moderately trained individuals involved in a variety of sports	8	Good
		SMIT	14	18.6 (1)	6	20.2 (3.1)					
		CON	9	20.9 (4.4)	7	19.7 (1.4)					
Dalzill 2014 [82]	NCT (retrospective)	HIIT					55	51.3 (8.7)	Metabolically healthy obese *NOTE: study included a metabolically unhealthy obese participant group (not extracted)*	6	Poor
Esbjörnsson Liljedahl 1996 [97]	NRCT	SIT	10	25 (range 21–29)	6	26 (range 23–30)			Recreationally active students at a College for Sports and Recreation Instructors	7	Good
		CON	5	24 (range 22–27)	5	24 (range 23–26)					
Fisher 2017 [96] + Hoffmann 2021 [73]	NCT	HIIT	8	30 (4.8) (range 26–34)	9	29 (6.3) (range 24–34)			Trained to well-trained competitive cyclists and triathletes, aged 18–40 y	8	Good
Gillen 2014 [83]	NCT	SIT	7	30 (10)	7	29 (9)			Overweight and obese but otherwise healthy adults	7	Good
Hiam 2021 [84]	NCT	HIIT	22	34.8 (7.0)	74	31.2 (8.2)			Healthy males and healthy pre-menopausal females	7	Good
Hirsch 2021 [72]	NCT	HIIT	10	36.80 (5.59)	9	36.67 (5.96)			Overweight and obese but otherwise healthy adults (non-smokers, < 150 min/wk of moderate exercise, < 2 d/wk of resistance training, and no HIIT in the previous 12 wk)	8	Fair
Lepretre 2009 [85]	NCT	HIIT	19	65.5 (5.4)	16	64.6 (3.7)	35	65.4 (4.9)	Healthy older adults	7	Fair
Liu 2021 [86]	RCT	SIT	8	20.5 (1.4)	8	20 (1.3)			Elite badminton players	8	Good
		CON	8	19.4 (1.5)	8	21.5 (2.2)					
Marterer 2020 [67]	NCT	HIIT	11	24 (3)	9	27 (5)	20	26 (4)	Healthy trained adults	6	Fair
Menz 2015 [87]	RCT	HIIT	5	25 (1)	14	28 (3)	19	27 (3)	Well-trained athletes ($\dot{V}$O_2max_ 63.7 ± 7.7 mL·kg^−1^·min^−1^. Normal exercise load: 9.5 ± 5.0 h/wk)	7	Good
		CON	3	23 (1)	13	25 (1)	16	24 (2)			
Metcalfe 2012 [88]	RCT	REHIT	8	24 (3)	7	26 (3)			Sedentary but healthy adults	8	Good
		CON	8	21 (1)	6	19 (1)					
Metcalfe 2016 [68]	NCT	REHIT	18	36 (9)	17	33 (9)			Sedentary but healthy adults	8	Good
Molina-Hidalgo 2020 [69]	QRCT	HIIT (0.0% alcohol beer)	7	23.4 (4)	8	25.4 (8.1)			Healthy adults *NOTE: study included HIIT* + *beer and HIIT* + *water and vodka groups (not extracted). Participants self-selected to training or non-training condition*	8	Good
		HIIT (water)	7	20.9 (2.7)	9	26.9 (7.6)					
		CON	7	19.9 (2.3)	7	20.1 (2.9)					
Mucci 2004 [70]	NCT	HIIT	10	18.5 (0.7)	12	18.4 (0.7)	22	18.5 (0.5)	Non-elite sportsmen and sportswomen	8	Good
Phillips 2017 [89]	NRCT	5-by-1 HIIT					136	36 (9)	Sedentary overweight and obese but otherwise healthy adults	6	Poor
		7-by-1 HIIT					40	37 (10)			
		CON					13	31 (11)			
Sawashita 2009 [90]	NCT	High-intensity interval walking	17	60.3 (SEM: 1.6)	6	65.2 (SEM: 1.8)	23	61.6 (SEM: 1.3)	Middle-aged and older (40–69 y), overweight and obese (BMI ≥ 23.6 kg/m^2^), Japanese adults *NOTE: study included a high-interval walking and mild calorie restriction group (not extracted)*	5	Poor
Scalzo 2014 [91]	NCT	SIT	10	22 (SEM: 1)	11	23 (SEM: 1)			Healthy recreationally active adults	7	Good
Schmitz 2019 [75]	NCT	Unmodified 4 × 30 s SIT	7		10		17	22.0 (1.6)	Moderately trained university students (Physical Education)	6	Fair
		Progressive 4–7 × 30 s SIT	8		9		17	21.1 (1.8)			
Schmitz 2020 [95]	NCT	4 × 30:30	14	23.1 (1.4)	7	22.1 (1.8)			Young healthy moderately trained university students (Physical Education)	5	Fair
		4 × 30:180	12	23.4 (4.4)	5	23.6 (1.3)					
Schubert 2017a [71] + Schubert 2017b [92]	QRCT	HIIT	7		5				Moderately active (> 120 min/wk of PA), healthy adults	6	Fair
		SIT	7		5						
		CON	3		3						
Søgaard 2018 [93] + Chrøis 2020 [81]	NCT	HIIT	11	63 (SEM 2)	11	63 (SEM 1)			Older sedentary adults (inclusion criteria were age 55–75 years)	7	Good
Støren 2017 [94]	NCT	HIIT	22		72		94	48.6 (18.2)	Healthy male and female matched for pre-test $\dot{V}$O_2max_ relative to age: typically, weekly low to moderate aerobic activity (0–2 h/wk)	6	Fair
Weber 2002 [74]	NCT	HIIT	7	22.7 (SEM 2.6)	7	23.7 (SEM 1.6)			Untrained males and females	8	Good

All values are reported as mean (standard deviation) unless otherwise stated

*AHRQ* categorization according to Agency for Healthcare Research and Quality criteria, *BMI* body mass index, *CON* control, *HIIT* high-intensity interval training, *NCT* non-controlled trial, *NOS* Newcastle–Ottawa Scale, *NRCT* non-randomized controlled trial, *PA* physical activity, *QRCT* quasi-randomized controlled trial (some self-selected groups), *RCT* randomized controlled trial, *REHIT* reduced-exertion HIIT, *SEM* standard error of the mean, *SIT* sprint interval training, *SMIT* supra-maximal interval training

**Table 2:** Summary of training protocols used within the included studies

Study	Session type	Exercise modality	Intervention length	Sessions/week or total	Interval training protocol	Session duration	Dose delivered vs dose received (fidelity of the intervention)
Astorino 2011 [76] + Astorino 2012 [66]	SIT	Cycle ergo	2–3 wk	6 × in total, separated by ≥ 2 d	Intervals: 4 × 30-s sprints Intensity: all-out ‘Wingate’ interval Recovery: 5-min passive	Not stated	All participants completed all requirements of the protocol
	CON	N/A	Not stated				
Bagley 2016 [77] + Bagley 2021 [78]	SIT	Cycle ergo	12 wk	3 × /wk	Intervals: 4 × 20-s sprints Intensity: ≈ 175% *V*O_2max_ Recovery: 2 min	≈ 10 min	Participants maintained a training log to record workloads during training sessions. No further details
Bornath 2022 [79]	SIT	Battling ropes	6 wk	3 × /wk	Intervals: 10 × 30-s Intensity: > 85% HR_max_ Recovery: 60 s	Not stated	Number of repetitions plus exercise intensity monitored during sessions (HR, RPE, and BLa)
Bostad 2021 [80]	SIT	Cycle ergo	12 wk	3 × /wk, separated by ≥ 1 d	Intervals: 2 × 20-s sprints Intensity: all-out Recovery: 2 min	≈ 10 min	28 ± 3 supervised sessions completed. No further details
Cicioni-Kolsky 2013 [98]	HIIT	Running on 200-m grass track	6 wk	2 × /wk	Intervals: 4 × 4 min → 5 × 4 min → 6 × 4 min (add interval every 2nd week) Intensity: 3000 m TT average pace Recovery: 1:1 work to rest ratio	Not stated	Only data of participants who completed all components of the training study were analyzed
	SMIT	Running on 200-m grass track	6 wk	3 × /wk	Intervals: 7 × 30-s sprints adding 1 × sprint interval each week Intensity: 130% of 3000 m TT average pace Recovery: 150 s passive recovery	Not stated	
Dalzill 2014 [82]	HIIT	Cycle ergo	9 mo	2–3 × /wk	Intervals: 15–30 s Intensity: 80% of maximal aerobic power Recovery: 15–30 s Sets: 2 × 10 min with 4 min passive recovery	≈ 60 min	Adherence to exercise training sessions was 3.0 ± 1.1 and 2.7 ± 1.0 sessions/week for metabolically healthy and unhealthy obese groups, respectively
Esbjörnsson Liljedahl 1996 [97]	SIT	Cycle ergo	4 wk	3 × /wk	Intervals: 3 × 30-s sprints Intensity: all-out ‘Wingate’ interval Recovery: 20 min	Not stated	There was no significant difference between the sexes in relative training intensity. No further details
Fisher 2017 [96] + Hoffmann 2021 [73]	HIIT	Cycle ergo	6 wk	1–3 × /wk 10 sessions total	Intervals: 10 × 90 s Intensity: 100% PPO Recovery: 60 s active recovery	Not stated	Training diaries were completed to ensure compliance and to compare between sexes. No between-sex differences were found for end HR, RPE or peak BLa measures across the HIIT sessions
Gillen 2014 [83]	SIT	Cycle ergo	6 wk	3 × /wk	Intervals: 3 × 20 s sprints Intensity: all-out Recovery: 2 min	10 min	Adherence to the training sessions was 100%. Relative peak and mean power output during first and last session did not differ between men and women
Hiam 2021 [84]	HIIT	Cycle ergo	4 wk	3 × /wk	Intervals: progression from 2 × 2 min → 16 × 2 min Intensity: 40–70% delta PPO—power at LT Recovery: 1 min at 60 W	Not stated	All participants trained under supervision. Intensity was individually determined from baseline graded exercise test. No further details
Hirsch 2021 [72]	HIIT	Cycle ergo	8 wk	2 × /wk	Intervals: Week 1: 6 × 1 min Week 2: 7 × 1 min Week 3: 8 × 1 min Week 4: 9 × 1 min Weeks 5–8: 10 × 1 min Intensity: 90% of max Wattage Recovery: 1-min rest	12–20 min	All training sessions were supervised one-on-one. Mean compliance to the HIIT protocol was 96 ± 9% for the entire 8 wk. Completion of at least 13 sessions was considered compliant
Lepretre 2009 [85]	HIIT	Cycle ergo	9 wk	2 × /wk	Intervals: 6 × 1 min Intensity: 2nd ventilatory threshold Recovery: 4 min at 1st ventilatory threshold	30 min	Details not reported
Lui 2021 [86]	SIT	Cycle ergo	8 wk	3 × /wk	Intervals: 5 × 30 s sprints Intensity: all-out Recovery: 5 min	Not stated	HR was monitored during training to obtain HR_max_, time spent in %HR_max_ zones and training impulse. Mean and peak HR, total and effective training times were similar for men and women
	Control	Running	8 wk	Not stated	Continuous ‘Fartlek’ running at 65–79% HR_max_	40 min	
Marterer 2020 [67]	HIIT	Cycle ergo	6 wk	Week 1–3: 3 × /wk Week 4–6: 4 × /wk	Intervals: 4 × 4 min Intensity: 85–95% HR_max_ Recovery: 2 min at 65–75% HR_max_	Not stated	Session one was supervised to reinforce the protocol. HR was recorded during sessions and monitored by the test coordinator. Participants who did not complete > 30 of 31 HIIT sessions were excluded from analysis
Menz 2015 [87]	HIIT	Running on 400-m track	3 wk	Week 1: 3 × /wk Week 2–3: 4 × /wk	Intervals: 4 × 4 min Intensity: 90–95% HR_max_ Recovery: 4 min active recovery	Not stated	Sessions were supervised, and training data were recorded in a logbook. HR and RPE were monitored during sessions. All participants completed 11 sessions, except for one who completed 10
Metcalfe 2012 [88]	SIT	Cycle ergo	6 wk	3 × /wk	Intervals: Session 1: 1 × 10 s Sessions 2–3: 2 × 10 s Sessions 4–9: 2 × 15 s Sessions 10–18: 2 × 20 s Intensity: all-out Recovery: 3–4 min low intensity	10 min in total	All training sessions were supervised. Mean adherence to the training program was 97%. Mean RPE from the whole training program was higher for women than men
Metcalfe 2016 [68]	SIT	Cycle ergo	6 wk	3 × /wk	Intervals: Session 1: 1 × 10 s Sessions 2–3: 2 × 10 s Sessions 4–9: 2 × 15 s Sessions 10–18: 2 × 20 s Intensity: all-out Recovery: 3–4 min low intensity	10 min in total	All training sessions were supervised. Mean adherence to the training programme was 98.5%. Mean RPE was 14 ± 2 with no significant differences between men and women
Molina-Hidalgo 2020 [69]	HIIT	Circuit training (e.g. horizontal rows, squats, push ups)	10 wk	2 × /wk Separated by > 48 h	Intervals: 2 × sets of 8 × exercises Intensity: 8–10 RPE Recovery (sets): time NER active recovery	40 min → 65 min	Participants that did not attend ≥ 80% of sessions were not included in the analysis. Training sessions were supervised in groups of ≈8 and participants were continuously encouraged to reach the specific target intensities
	CON	N/A					
Mucci 2004 [70]	HIIT	Running	8 wk	3 × /wk	Sessions (each wk) Mon: 2 × sets of 13 × 5-s sprint at V_max_/20-s recovery Wed: 2 × sets of 15 × 10 s at vVO_2max_ + 40% (V_max_ − vVO_2max_)/10-s recovery Fri: 2 × sets of 6 × 30 s at vVO_2max_ + 40% (V_max _− vVO_2max_)/90-s recovery Recovery (sets): 5 min	Not stated	The target running speeds were achieved by setting a covering distance during the work time defined in each training session (i.e., 5, 10, or 30 s). Participants who did not regularly attend sessions were excluded
Phillips 2017 [89]	5-by-1 HIIT	Cycle ergo	6 wk	3 × /wk	Intervals: 5 × 1 min Intensity: 100% VO_2max_ Recovery: 90 s	Not stated	Sessions were fully supervised. No further details
	7-by-1 HIIT	Cycle ergo	6 wk	3 × /wk	Intervals: 7 × 1 min Intensity: 100% VO_2max_ increasing 10% every 2 wk Recovery: 90 s	Not stated	
	CON	N/A	6 wk				
Sawashita 2009 [90]	HIIT	Walking	16 wk	≥ 4 × /wk	Intervals: 5 × 3 min Intensity: 70–85% VO_2max_ Recovery: 3 min ≈40% VO_2max_	Not stated	Steps and intensity were monitored with an accelerometer with audio cues to signal the change in intensity during training. If targets were not met from accelerometer data, trainers encouraged participants to increase their efforts
Scalzo 2014 [91]	SIT	Cycle ergo	3 wk	9 sessions total Separated by 1–2 d	Intervals: 4–8 × 30 s Intensity: all-out Recovery: 4-min active recovery	Not stated	Details not reported
Schmitz 2019 [75]	HIIT	Running	4 wk	3 × /wk Separated by ≥ 1 d	Intervals: 4 × 30 s Intensity: all-out Recovery: 30-s active recovery	Not stated	The first and last training session were supervised and running speed was recorded. Reported adherence to training was 92.2 ± 12.35% for the HIIT group and 91.7 ± 11.28% for the proHIIT group
	ProHIIT	Running	4 wk	3 × /wk Separated by ≥ 1 d	Intervals: 4 × 30 s increasing by 1 interval each week → 7 × 30 s Intensity: all-out Recovery: 30-s active recovery	Not stated	
Schmitz 2020 [95]	SIT	Running on 200-m indoor track	4 wk	2 × /wk Separated by ≥ 2 d	Intervals: 4 × 30 s Intensity: all-out Recovery: 3-min active recovery	Not stated	Participants who completed < 6 of 8 training sessions were excluded from the analysis. Training sessions were controlled by at least two experienced trainers
	SIT	Running on 200-m indoor track	4 wk	2 × /wk Separated by ≥ 2 d	Intervals: 4 × 30 s Intensity: all-out Recovery: 30-s active recovery	Not stated	
Schubert 2017a [71] + Schubert 2017b [92]	HIIT	Cycle ergo	4 wk	3 × /wk	Intervals: Wk 1–2: 2 × 90 s; Wk 3–4: 4 × 90 s Intensity: 90% PPO Recovery: 1-min active recovery (10% PPO)	16–20 min	All participants completed all 12 sessions over 4 wk. Average HR during sessions were monitored to determine intensity
	SIT	Cycle ergo	4 wk	3 × /wk	Intervals: 3 × 20 s Intensity: all-out Recovery: 2-min active recovery (10% PPO)	10–15 min	
	CON	N/A	4 wk				
Søgaard 2018 [93] + Chrøis 2020 [81]	HIIT	Cycle ergo	6 wk	3 × /wk	Intervals: 5 × 1 min Intensity: 85% VO_2max_ increasing by 10% each interval Recovery: 90-s active recovery (25 W)	Not stated	All sessions were completed by 18 participants. Three men missed one session and one woman missed two sessions. Men and women trained at a similar load
Støren 2017 [94]	HIIT	Treadmill or cycle ergo	8 wk	3 × /wk	Intervals: 4 × 4 min Intensity: 90–95% of HR_max_ Recovery: 3-min active recovery at 70% HR_max_	Not stated	Training sessions were supervised. HR was monitored to verify target intensity. Mean compliance to the intervention was 92% ± 4%, with no significant differences between men and women
Weber 2002 [74]	HIIT	Cycle ergo	8 wk	3 × /wk	Intervals: 3 × 2 min Intensity: 85% → 100% of the workload = 120% VO_2peak_ Recovery: 6 min	Not stated	Peak HR recorded during training at weeks 1, 4 and 8 was not different between men and women

*BLa* blood lactate concentration, *CON* non-exercise control or comparison group, *HIIT* high-intensity interval training, *HR* heart rate, *HR*_*max*_ maximal heart rate, *LT* lactate threshold, *N/A* not applicable/no intervention, *NER* not explicitly reported, *PPO* peak power output on incremental test, *proHIIT* progressive HIIT, *RPE* rating of perceived exertion, *SIT* sprint interval training, *SMIT* supra-maximal interval training, *TT* time trial, *V*_*max*_ maximal running velocity, *VO*_*2max*_ maximal oxygen uptake, *VO*_*2peak*_ peak oxygen uptake, *vVO*_*2max*_ running velocity at VO_2max_, → denotes progressive overload throughout training intervention

**Table 3:** Summary of meta-analyses of $\dot{V}$O_2max_, peak power output from incremental testing, and work at anaerobic threshold, primary analysis, relative and absolute $\dot{V}$O_2max_, and sub-groupings by participant characteristics

Outcome	Sub-group		Within-group effects						Heterogeneity			Between-group difference		
			*n*_trials_	*n*_participants_	%Δ (*σ*)	*g*	95% CI	*p*	*I*^2^ (%)	*Q*	*p* (*Q*)	*Q*	*df*	*p*
Men vs women, total														
$\dot{V}$O_2max_		*Women*	19	265	11.16 (7.39)	0.57	(0.44–0.69)	**0.000**	62.14	47.54	**0.000**			
		*Men*	19	273	10.90 (5.75)	0.57	(0.42–0.72)	**0.000**	78.80	84.91	**0.000**	0.00	1	0.965
PPO		*Women*	8	99	11.16 (5.99)	0.56	(0.32–0.80)	**0.000**	75.84	28.97	**0.000**			
		*Men*	8	143	8.22 (5.09)	0.41	(0.22–0.59)	**0.000**	62.06	18.45	**0.010**	1.05	1	0.304
Work_AT_		*Women*	5	79	8.07 (6.55)	0.38	(0.13–0.64)	**0.001**	31.26	5.82	0.213			
		*Men*	5	123	7.09 (7.17)	0.38	(0.11–0.64)	**0.002**	29.85	5.70	0.223	0.00	1	0.964
Men vs women, relative $\dot{V}$O_2max_														
$\dot{V}$O_2max_		*Women*	16	205	11.35 (7.70)	0.57	(0.43–0.71)	**0.000**	63.27	40.84	**0.000**			
		*Men*	16	249	9.88 (4.92)	0.55	(0.38–0.71)	**0.000**	80.73	77.85	**0.000**	0.04	1	0.845
Men vs women, absolute $\dot{V}$O_2max_														
$\dot{V}$O_2max_		*Women*	7	87	10.33 (6.30)	0.56	(0.28–0.83)	**0.000**	80.33	30.50	**0.000**			
		*Men*	7	91	11.34 (6.28)	0.51	(0.32–0.70)	**0.000**	57.35	14.07	**0.029**	0.06	1	0.805
Men vs women by baseline training status														
$\dot{V}$O_2max_	Untrained	*Women*	11	164	13.22 (8.49)	0.63	(0.48–0.78)	**0.000**	56.81	23.15	**0.010**			
		*Men*	11	114	13.86 (5.23)	0.66	(0.48–0.85)	**0.000**	68.34	31.59	**0.000**			
	Moderately trained	*Women*	5	72	8.40 (6.12)	0.33	(0.15–0.51)	**0.000**	34.48	6.11	0.191			
		*Men*	5	130	4.37 (1.68)	0.18	(0.09–0.28)	**0.000**	0.00	3.39	0.494			
	Well trained	*Women*	3	29	8.23 (1.20)	0.72	(0.41–1.04)	**0.000**	23.73	2.62	0.269			
		*Men*	3	29	10.92 (1.14)	0.86	(0.86–0.87)	**0.000**	0.00	0.00	1.00			
	All groups											48.74	5	**0.000**
PPO	Untrained	*Women*	4	44	13.99 (6.73)	0.77	(0.36–1.18)	**0.000**	79.57	14.68	**0.002**			
		*Men*	4	41	12.23 (3.61)	0.60	(0.31–0.89)	**0.000**	54.58	6.61	**0.086**			
	Moderately trained	*Women*	2	36	9.96 (5.25)	0.35	(0.01–0.68)	**0.036**	54.31	2.19	0.139			
		*Men*	2	84	5.86 (0.65)	0.27	(0.14–0.41)	**0.000**	0.00	1.00	0.317			
	Well trained	*Women*	2	19	6.71 (3.67)	0.37	(0.26–0.48)	**0.000**	0.00	0.25	0.618			
		*Men*	2	18	2.56 (1.52)	0.17	(0.15–0.19)	**0.000**	0.00	0.01	0.927			
	All groups											11.04	5	**0.051**
Men versus women by group mean age														
$\dot{V}$O_2max_	Under 30 y	*Women*	10	93	9.49 (3.82)	0.63	(0.47–0.79)	**0.000**	35.93	14.05	0.121			
		*Men*	10	95	11.54 (5.65)	0.71	(0.54–0.89)	**0.000**	49.67	17.88	**0.037**			
	30–45 y	*Women*	5	81	10.63 (6.74)	0.39	(0.20–0.57)	**0.000**	52.12	8.35	**0.079**			
		*Men*	5	134	8.04 (6.78)	0.40	(0.04–0.75)	**0.000**	88.73	35.51	**0.000**			
	Over 45 y	*Women*	4	91	15.99 (13.52)	0.68	(0.35–1.00)	**0.000**	72.45	10.89	**0.012**			
		*Men*	4	44	12.88 (4.64)	0.49	(0.21–0.77)	**0.000**	52.66	6.34	**0.096**			
	All groups											8.60	5	0.126

*p*(*Q*) significance set at < 0.10; effect size (*g*) significance set at *p* = 0.05. Bolded *p* values indicate statistical significance

*%Δ* percentage change (unweighted mean across studies), *σ* standard deviation (of %Δ), *CI* confidence interval, *df* degrees of freedom, *g* effect size (Hedges’ *g*), *I*^*2*^ Higgins *I*^*2*^, *PPO* peak power output from incremental exercise testing, *Q* Cochran’s *Q* (sum of squares), $\dot{V}$*O*_*2max*_ maximal oxygen uptake, *Work*_*AT*_ threshold power (inclusive of lactate and ventilatory thresholds)

**Table 4:** Summary of meta-analyses of $\dot{V}$O_2max_ and peak power output from incremental testing, sub-groupings by intervention characteristics

Outcome	Sub-group			Within-group effects						Heterogeneity			Between-group difference		
				*n*_trials_	*n*_participants_	%Δ (*σ*)	*g*	95% CI	*p*	*I*^2^ (%)	*Q*	*p* (*Q*)	*Q*	*df*	*p*
Men vs women, HIIT versus SIT															
$\dot{V}$O_2max_	HIIT		*Women*	11	177	11.00 (9.29)	0.52	(0.34–0.71)	**0.000**	76.06	41.77	**0.000**			
			*Men*	11	172	11.20 (5.54)	0.60	(0.37–0.82)	**0.000**	85.98	71.32	**0.000**			
	SIT		*Women*	9	96	10.86 (4.19)	0.59	(0.46–0.71)	**0.000**	0.00	6.77	0.562			
			*Men*	9	93	9.82 (6.32)	0.46	(0.27–0.65)	**0.000**	52.58	16.87	**0.040**			
	All groups												1.36	3	0.716
PPO	HIIT		*Women*	7	131	8.26 (5.50)	0.48	(0.28–0.68)	**0.000**	69.41	19.61	**0.003**			
			*Men*	7	85	10.10 (5.69)	0.41	(0.20–0.62)	**0.000**	66.08	17.69	**0.007**			
	SIT		*Women*	2	14	7.16 (1.14)	0.90	(0.14–1.66)	**0.011**	73.79	3.82	**0.051**			
			*Men*	2	12	16.09 (2.71)	0.33	(− 0.01 to 0.68)	**0.033**	9.94	1.11	0.292			
	All groups												2.34	3	0.506
Men vs women by intervention length															
$\dot{V}$O_2max_	≤ 4 weeks		*Women*	4	55	5.82 (2.37)	0.25	(0.11–0.39)	**0.000**	0.00	2.20	0.532			
			*Men*	4	106	3.97 (1.63)	0.16	(0.05–0.27)	**0.003**	0.00	2.51	0.474			
	5–9 weeks		*Women*	10	109	9.39 (4.23)	0.55	(0.40–0.70)	**0.000**	29.97	12.85	0.169			
			*Men*	10	103	11.24 (3.64)	0.67	(0.50–0.85)	**0.000**	58.70	21.79	**0.010**			
	≥ 10 weeks		*Women*	5	101	18.98 (9.42)	0.79	(0.63–0.94)	**0.000**	19.55	4.97	0.290			
			*Men*	5	64	15.76 (6.27)	0.66	(0.32–1.00)	**0.000**	80.41	20.42	**0.000**			
	All groups												59.35	5	**0.000**

*p*(*Q*) significance set at < 0.10; effect size (*g*) significance set at *p* = 0.05. Bolded *p* values indicate statistical significance

*%Δ* percentage change (unweighted mean across studies), *σ* standard deviation (of %Δ), *CI* confidence interval, *df* degrees of freedom, *g* effect size (Hedges’ *g*), *I*^*2*^ Higgins *I*^*2*^, *PPO* peak power output from incremental exercise testing, *Q* Cochran’s *Q* (sum of squares), $\dot{V}$*O*_*2max*_ maximal oxygen uptake, *Work*_*AT*_ threshold power (inclusive of lactate and ventilatory thresholds)

**Table 5:** Summary of outcomes and results of included studies: maximal oxygen uptake

References	Measurement	Outcome (units)	Sex, baseline	Δ M	Δ F	Interaction
Maximal oxygen uptake						
Astorino 2011 [76] + Astorino 2012 [66]	Cycle ergo test, indirect calorimetry	$\dot{V}$O_2max_ (mL/kg/min)^a^	NS	+	+	NS
		$\dot{V}$O_2max_ (L/min)	M > F (*p* < 0.05)	+ (5.9 ± 3.9%)	+ (6.8 ± 7.1%)	NS (*p* = 0.58)
Bagley 2016 [77] + Bagley 2021 [78]	Cycle ergo test, indirect calorimetry	$\dot{V}$O_2max_ (ml/kg/min)^a^	(*p* = 0.005)	+ (6.0%)	+ (18.7%)	F > M (*p* = 0.049)
		$\dot{V}$O_2max_ (L/min)^a^	(*p* < 0.001)	+	+	F > M (*p* = 0.009)
Bornath 2022 [79]	Arm cycle test, indirect calorimetry (Astrand protocol)	$\dot{V}$O_2max_—arms (mL/kg/min), 3 wk	NER	+ (ES: < 0.45; *p* < 0.004)	+ (ES: 0.67; *p* < 0.001)	NER
		$\dot{V}$O_2max_ –arms (mL/kg/min), 6 wk	NER	+ (ES: 0.66; *p* < 0.001)	+ (ES: 0.48; *p* = 0.001)	NER
Bostad 2021 [80]	Cycle ergo test, indirect calorimetry	$\dot{V}$O_2peak_ (L/min), 2 wk	NER	+ (*p* = 0.03)	NS	M > F (*p* < 0.01)
		$\dot{V}$O_2peak_ (L/min), 6 wk		+ (*p* < 0.01)	+ (*p* < 0.01)	NS
		$\dot{V}$O_2peak_ (L/min), 12 wk^a^		+ (*p* < 0.01)	+ (*p* < 0.01)	NS
Dalzill 2014 [82]	Incremental treadmill test	$\dot{V}$O_2peak_ (METs)^a^	M > F (*p* < 0.001)	+ (*p* < 0.0001)	+ (*p* < 0.0001)	NS
Gillen 2014 [83]	Cycle ergo test, indirect calorimetry	$\dot{V}$O_2peak_ (mL/kg/min)^a^ $\dot{V}$O_2peak_ (L/min; baseline only)	NS (*p* ≤ 0.05)	+ (12%; *p* < 0.001)	+ (12%; *p* < 0.001)	NS
Hiam 2021 [84]	Cycle ergo test, indirect calorimetry	$\dot{V}$O_2peak_ (mL/kg/min)^a^	M > F (*p* = 0.04)	NER	NER	NS (*p* = 0.48)
Hirsch 2021 [72]	Cycle ergo test, indirect calorimetry	$\dot{V}$O_2peak_ (mL/kg/min), 0–4 wk	M > F (*p* = 0.03)	NS (6.8%)	NS (− 0.5%)	NS
		$\dot{V}$O_2peak_ (mL/kg/min), 4–8 wk		NS (12.7%)	+ (16.6%; *p* < 0.05)	NS
		$\dot{V}$O_2peak_ (mL/kg/min), 0–8 wk ^a^		+ (19.5%; *p* < 0.05)	+ (16.1%; *p* < 0.05)	NS
Lepretre 2009 [85]	Cycle ergo test, indirect calorimetry	$\dot{V}$O_2peak_ (mL/kg/min)^a^	M > F (*p* < 0.05)	+ (14.9%; *p* < 0.001)	+ (14.5%; *p* < 0.001)	NS
Liu 2021 [86]	Treadmill test, indirect calorimetry	$\dot{V}$O_2max_ (mL/kg/min)^a^	NER	+ (ES: 1.14; *p* < 0.05)	+ (ES: 1.28; *p* < 0.05)	NER
Marterer 2020 [67]	Cycle ergo test, indirect calorimetry	$\dot{V}$O_2max_—legs (mL/kg/min)^a^	NER	+ (*p* < 0.001)	+ (*p* = 0.002)	NS (*p* = 0.088)
		$\dot{V}$O_2max_—legs (mL/min)^a^	NER	+ (9.96%; *p* < 0.001)	+ (7.95%; *p* = 0.002)	M > F (*p* = 0.007)
	Arm cycle test, indirect calorimetry	$\dot{V}$O_2max_—arms (mL/kg/min)	NER	NS	NS	NS (*p* = 0.267)
		$\dot{V}$O_2max_—arms (mL/min)	NER	+ (*p* = 0.031)	NS	NS (*p* = 0.134)
Menz 2015 [87]	Treadmill test, indirect calorimetry	$\dot{V}$O_2max_ (mL/kg/min)	NER	NER	NER	NS, data pooled
		$\dot{V}$O_2max_ (mL/min)	NER	NER	NER	NS, data pooled
Metcalfe 2012 [88]	Cycle ergo test, indirect calorimetry	$\dot{V}$O_2peak_ (mL/kg/min)^a^	M > F (*p* < 0.001)	+ (15.0%; *p* < 0.01)	+ (12.0%; *p* < 0.01)	NS
Metcalfe 2016 [68]	Cycle ergo test, Douglas bag method	$\dot{V}$O_2max_ (mL/kg/min)^a^	M > F (*p* = 0.010)	+ (*p* < 0.001)	+ (*p* < 0.001)	NS (*p* = 0.926)
		$\dot{V}$O_2max_ (L/min)^a^	M > F (*p* < 0.001)	+ (*p* < 0.001)	+ (*p* < 0.001)	NS (*p* = 0.402)
Molina-Hidalgo 2020 [69]	Treadmill test, indirect calorimetry (modified Balke)	$\dot{V}$O_2max_ (mL/kg/min)^a^	NER	+ (*p* < 0.05)	+ (*p* < 0.05)	NER
		$\dot{V}$O_2max_ (mL/min)^a^	NER	+ (*p* < 0.05)	+ (*p* < 0.01)	NER
Mucci 2004 [70]	Multi-stage running test, portable gas analysis	$\dot{V}$O_2max_ (mL/kg/min)^a^	M > F (*p* < 0.05)	+ (10.0%; *p* < 0.001)	+ (7.8%; *p* < 0.01)	NS
Phillips 2017 [89]	Cycle ergo test, indirect calorimetry	$\dot{V}$O_2max_ (mL/kg/min; Δ% only)	NER	NER	NER	NS
		$\dot{V}$O_2max_ (L/min; 5-by-1 HIIT)	NER	+ (0.32 ± 0.3 L/min)	+ (0.19 ± 0.2 L/min)	M > F (*p* < 0.001)
Sawashita 2009 [90]	Estimated from graded walking test, triaxial accelerometer and ECG	$\dot{V}$O_2peak_ (mL/kg/min)^a^	NER	+ (< 0.001)	+ (< 0.001)	NS
Scalzo 2014 [91]	Cycle ergo test, indirect calorimetry	$\dot{V}$O_2max_ (mL/kg/min)^a^	NS	+ (0.6 mL/kg/min; *p* = 0.002)	+ (3.0 mL/kg/min; *p* = 0.002)	NS (*p* = 0.287)
Schmitz 2020 [95]	Estimated from maximal running speed (Léger and Boucher equation)	$\dot{V}$O_2max_ (mL/kg/min)	NER	NER	NER	NER, baseline only
Schubert 2017a [71] + Schubert 2017b [92]	Cycle ergo test, indirect calorimetry	$\dot{V}$O_2max_ (mL/kg/min)^a^ $\dot{V}$O_2max_ (L/min; pooled only)	NER	NER	NER	NER, individual data presented
Søgaard 2018 [93] + Chrøis 2020 [81]	Cycle ergo test, indirect calorimetry	$\dot{V}$O_2max_ (mL/kg/min)^a^	M > F (*p* < 0.012)	+ (*p* = 0.024)	+ (*p* = 0.024)	NS (*p* = 0.277)
		$\dot{V}$O_2max_ (mL/min)^a^	M > F (*p* < 0.001)	+ (*p* = 0.030)	+ (*p* = 0.030)	NS (*p* = 0.473)
Støren 2017 [94]	Treadmill or cycle ergometer test, indirect calorimetry	$\dot{V}$O_2max_ (mL/kg/min; pooled only)	NER	+ (4.1 ± 2.5 mL/kg/min; *p* < 0.001)	+ (4.2 ± 2.5 mL/kg/min; *p* < 0.001)	NS (*p* = 0.300)
		$\dot{V}$O_2max_ (L/min; pooled only)	NER	+ (10.8 ± 8.1%; *p* < 0.001)	+ (13.8% ± 10.0%; *p* < 0.001)	NER
Weber 2002 [74]	Cycle ergo test, indirect calorimetry	$\dot{V}$O_2max_ (mL/kg/min, baseline only)	M > F (*p* < 0.05)	NER	NER	NER
		$\dot{V}$O_2peak_ (L/min)^a^	M > F (*p* < 0.001)	+ (7.9 ± 2.0%; *p* < 0.01)	NS (2.9 ± 1.5%)	M > F (*p* < 0.05)

Δ indicates change (i.e., pre-post), + denotes a significant increase; *ES* effect size, *F* women/females, *M* men/males, *MET* metabolic equivalent, *NER* not explicitly reported, *NS* not significant, $\dot{V}$*O*_*2max*_ maximal oxygen uptake, $\dot{V}$*O*_*2peak*_ peak oxygen uptake

^a^Included in meta-analyses

**Table 6:** Summary of outcomes and results of included studies, peak power output from incremental testing and threshold power

References	Measurement	Outcome (units)	Sex, baseline	Δ M	Δ F	Interaction
Peak power output from incremental testing						
Bostad 2021 [80]	Cycle ergo test, indirect calorimetry	*W*_peak_ (W; for ExRx only)	NER	NER	NER	NER
Fisher 2017 [96] + Hoffmann 2021 [73]	Cycle ergo test, earlobe capillary blood sampling	Absolute PPO (W)^a^	M > F (*p* < 0.001)	+ (*p* = 0.05)	+ (*p* = 0.05)	NS
		Relative PPO (W·kg^−1^ and W·kg^−0.32^)	M > F (*p* < 0.001)	+ (*p* = 0.04)	+ (*p* = 0.04)	NS
Gillen 2014 [83]	Cycle ergo test, indirect calorimetry	Maximal workload (W; baseline)	M > F (*p* ≤ 0.05)	+ (14%; *p* < 0.001)	+ (14%; *p* < 0.001)	NS
		Relative PPO (W/kg FFM)^a^	NER	+ (0.9 W/kg FFM; *p* ≤ 0.05)	+ (1.8 W/kg FFM; *p* ≤ 0.05)	NER
		Relative MPO (W/kg FFM)	NER	+ (1.6 W/kg FFM; *p* ≤ 0.05)	+ (3.0 W/kg FFM; *p* ≤ 0.05)	NER
Hiam 2021 [84]	Cycle ergo test, indirect calorimetry	PPO (W/kg)^a^	M > F (*p* = 0.030)	NER	NER	NS (*p* = 0.650)
Lepretre 2009 [85]	Cycle ergo test, indirect calorimetry	MTP (W)^a^	M > F (*p* ≤ 0.05)	+ (*p* < 0.001)	+ (*p* < 0.001)	NS
Marterer 2020 [67]	Cycle ergo or arm cycle test, earlobe capillary blood sampling	PPO—legs (W)^a^	NER	+ (*p* = 0.033)	+ (*p* = 0.026)	NS (*p* = 0.562)
		PPO—arms (W)	NER	NS (*p* = 0.176)	NS (*p* = 0.498)	NS (*p* = 0.563)
Phillips 2017 [89]	Incremental cycle ergo test	PPO/W_max_ (W; pooled only)	NER	NER	NER	NER
Schubert 2017a [71] + Schubert 2017b [92]	Cycle ergo test, highest workload completed for 60 s	PPO (W)^a^	NER	NER	NER	NER, individual data presented
Søgaard 2018 [93] + Chrøis 2020 [81]	Cycle ergo test, indirect calorimetry	Maximum workload (W)^a^	M > F (*p* < 0.001)	+ (31 W; *p* < 0.001)	NS (9 W)	M > F (*p* = 0.004)
Støren 2017 [94]	Treadmill or cycle ergometer test, indirect calorimetry	Work performance (W)	NER	+ (24.5 ± 34.4%; *p* < 0.001)	+ (23.8 ± 43.7%; *p* < 0.001)	NS (*p* = 0.980)
Weber 2002 [74]	Cycle ergo test, indirect calorimetry	PPO (W)^a^	M > F (*p* < 0.001)	+ (10.7 ± 2.0%; *p* < 0.01)	+ (11.2 ± 1.1%; *p* < 0.01)	NS
Threshold power						
Fisher 2017 [96] + Hoffmann 2021 [73]	Cycle ergo test, earlobe capillary blood sampling	LT_2_ power output (W)^a^	M > F (*p* < 0.001)	NS	NS	NS
Hiam 2021 [84]	Cycle ergo test, indirect calorimetry	Power output at LT (W/kg)^a^	M > F (*p* = 0.040)	NER	NER	NS (*p* = 0.410)
Lepretre 2009 [85]	Cycle ergo test, indirect calorimetry	Power at VT_1_ (W)	M > F (*p* ≤ 0.05)	+ (29.0%; *p* < 0.001)	+ (32.5% *p* < 0.001)	NS
		Power at VT_1_ (%MTP)	NS	+ (12.0%; *p* < 0.001)	+ (9.0%; *p* < 0.001)	NS
		Power at VT_2_ (W)^a^	M > F (*p* ≤ 0.05)	+ (*p* < 0.001)	+ (*p* < 0.001)	NS
		Power at VT_2_ (%MTP)	M > F (*p* ≤ 0.05)	NS	NS	NS
Marterer 2020 [67]	Cycle ergo or arm cycle test, earlobe capillary blood sampling *LT defined as 4 mmol/L of BLa*	Power output at LT—legs (W/kg)	NER	NS (*p* = 0.291)	NS (*p* = 0.059)	NS (*p* = 0.878)
		Power output, at LT—legs (W)^a^	NER	NS (*p* = 0.301)	+ (*p* = 0.054)	NS (*p* = 0.962)
		Power output at LT—arms (W/kg)	NER	NS (*p* = 0.714)	NS (*p* = 0.195)	NS (*p* = 0.384)
		Power output, at LT—arms (W)	NER	NS (*p* = 0.580)	NS (*p* = 0.182)	NS (*p* = 0.178)
Schmitz 2019 [75]	Incremental running test, earlobe capillary blood sampling	Speed at LT (m/s)^a^, pooled HIIT + proHIIT	NER	+ (0.13 m/s)	+ (0.10 m/s)	NS (*p* = 0.09)
Schmitz 2020 [95]	Incremental running test, capillary blood sampled rest + immediate post	Speed at LT (km/h; baseline only)	M > F (*p* < 0.001)	NER	NER	NER

+ denotes a significant increase; Δ denotes change (i.e., pre-post); *BLa* blood lactate concentration, *ExRx* exercise prescription, *F* women/females, *FFM* fat free mass, *LT* lactate threshold, *LT*_*2*_ second lactate threshold, *M* men/males, *MTP* maximal tolerated power, *NER* not explicitly reported, *NS* not significant, *PPO* peak power output, *VT*_*1*_ first ventilatory threshold, *VT*_*2*_ second ventilatory threshold, *HIIT* high-intensity interval training, *proHIIT* progressive HIIT, *W* Watts, *W*_*max*_ maximum Watts, *W*_*peak*_ peak Watts

^a^Included in meta-analyses

**Table 7:** Summary of outcomes and results of included studies, additional cardiorespiratory and performance outcomes and measures of fatigue

References	Measurement	Outcome (units)	Sex, baseline	Δ M	Δ F	Interaction
Threshold oxygen uptake						
Hirsch 2021 [72]	Cycle ergo test, indirect calorimetry	VT (L/min), 0–4 weeks	Main effect	NS	NS	NS
		VT (L/min), 4–8 weeks	(*p* = 0.004)	NS	+ (*p* < 0.05)	NS
		VT (L/min), 0–8 weeks		+ (*p* < 0.05)	NS	NS
Lepretre 2009 [85]	Cycle ergo test, indirect calorimetry	VT_1_ (%$\dot{V}$O_2peak_)	M > F (*p* = 0.054)	+ (*p* = 0.011)	+ (*p* = 0.011)	NS
		VT_2_ (%$\dot{V}$O_2peak_)	NS (*p* = 0.362)	NS (*p* = 0.362)	NS (*p* = 0.362)	NS
Liu 2021 [86]	Treadmill test, indirect calorimetry	VT (%$\dot{V}$O_2max_)	NER (M: 74.0%; F: 75.0%)	+ (11.0%; *p* < 0.05)	+ (7.0%; *p* < 0.05)	NER
		VT (mL/min)	NER	+ (19.0%; *p* < 0.05)	+ (11.0%; *p* < 0.05)	NER
Marterer 2020 [67]	Cycle ergo test, earlobe capillary blood sampled end of each stage	$\dot{V}$O_2_ at LT—legs (mL/min)	NER	NS (*p* = 0.097)	NS (*p* = 0.058)	NS (*p* = 0.427)
		$\dot{V}$O_2_ at LT—legs (mL/min/kg)	NER	NS (*p* = 0.088)	NS (*p* = 0.249)	NS (*p* = 0.960)
	Arm cycle test, earlobe capillary blood sampled end of each stage *LT defined as 4mmol/L of BLa*	$\dot{V}$O_2_ at LT—arms (mL/min)	NER	NS (*p* = 0.859)	NS (*p* = 0.191)	NS (*p* = 0.296)
		$\dot{V}$O_2_ at LT—arms (mL/min/kg)	NER	NS (*p* = 0.844)	NS (*p* = 0.296)	NS (*p* = 0.465)
Wingate power						
Astorino 2011 [76] + Astorino 2012 [66]	Wingate test (repeated; two tests performed with 5 min of active recovery in between)	Maximal power (W/kg; pooled)	M > F (*p* < 0.05)	+ (10.5%; *p* < 0.05)	+ (9.1%; *p* < 0.05)	NS
		Mean power (W/kg; pooled)	M > F (*p* < 0.05)	+ (10.4%; *p* < 0.05)	+ (10.9%; *p* < 0.05)	NS
		Minimum power (W/kg; pooled)	NS	+ (14.9%; *p* < 0.05)	+ (9.5%; *p* < 0.05)	NS
Esbjörnsson Liljedahl 1996 [97]	Wingate test (repeated; three tests performed with a 20-min rest in between)	Maximal power (W), 1st test	NER	NS	+ (6.0%; *p* < 0.01)	NS (*p* < 0.06)
		Maximal power (W), 2nd test	NER	NS	NS	NS
		Maximal power (W), 3rd test	NER	NS	NS	NS
		Mean power (W), 1st test	NER	NS	+ (10.0%; *p* < 0.01)	F > M (*p* < 0.05)
		Mean power (W), 2nd test	NER	NS	+ (6.0%; *p* < 0.05)	F > M (*p* < 0.05)
		Mean power (W), 3rd test	NER	NS	+ (4.0%; *p* < 0.05)	NS
		Absolute power difference between M and F, mean of 3 tests	M > F (47.0%)	N/A	N/A	Post: M > F (38.0%; *p* < 0.03)
Fatigue and speed decrement						
Astorino 2011 [76] + Astorino 2012 [66]	Wingate test (repeated; two tests performed with 5 min of active recovery in between)	Fatigue index (%; pooled)	M > F (M: 55.2%; F: 47.3%; *p* < 0.05)	NS (− 3.3%)	NS (0.0%)	NS
Scalzo 2014 [91]	The mean power from sprints during sessions 1 and 9	Fatigue index (%), session 1–9	NS (M: 28.0%; F: 13.0%; *p* < 0.066)	– (12%)	– (3%)	NS (*p* = 0.066)
Schmitz 2020 [95]	Incremental running test	Maximal speed (km/h; baseline only) Repeated sprint speed decrement, 4 × 30:30 Repeated sprint speed decrement, 4 × 30:180	M > F (*p* < 0.001)	NER	NER	NER
			NER	NS	– (3.4%; *p* = 0.004)	F > M (*p* = 0.0038)
			NER	NS	NS	NS
Fisher 2017 [96] + Hoffmann 2021 [73]	Cycle ergo test	Incremental time to fatigue (min)	F > M (*p* < 0.01)	+ (1.8%; *p* = 0.01)	+ (5.1%; *p* = 0.01)	NS
Weber 2002 [74]	Timed cycling test to fatigue at 120% $\dot{V}$O_2peak_	Time to fatigue (s), Δ pre-mid training	NS	+ (30.6 ± 3.4%; *p* < 0.01)	+ (29.0 ± 3.2%; *p* < 0.01)	NER
		Time to fatigue (s), Δ mid-post training	NS	+ (12.4 ± 3.7%; *p* < 0.05)	+ (9.6 ± 3.9%; *p* < 0.05)	NER
Time trials, maximal performance, and sprint ability						
Bostad 2021 [80]	Average power output from all sprints and all training sessions	Sprint power output (%*W*_peak_)	N/A	N/A	N/A	NS (M: 237% W_peak_; F: 222% W_peak_; *p* = 0.25)
	2 kJ/kg body weight cycle ergo time trial (distance NER)	Time trial completion, 2 wk	NER	– (*p* < 0.01)	– (*p* < 0.01)	NER, pooled only
		Time trial completion, 6 wk		– (*p* = 0.14)	– (*p* = 0.14)	
		Time trial completion, 12 wk		– (*p* < 0.01)	– (*p* < 0.01)	
Cicioni-Kolsky 2013 [98]	Maximal, 640 m repeated sprint ability test (sum of six sprints)	40-m maximal sprint (s), HIIT	NER	NS (1.28%; *p* = 0.091)	– (1.95%; *p* = 0.013)	NS (HIIT + SMIT, *p* = 0.680)
		40-m maximal sprint (s), SMIT	NER	– (2.99%; *p* = 0.010)	– (3.73%; *p* < 0.001)	
	2 kJ/kg body weight cycle ergometer time trial	Sum of six sprints (s), HIIT	NER	– (2.86%; *p* = 0.020)	– (4.90%; *p* = 0.001)	NS (HIIT + SMIT, *p* = 0.593) F > M (HIIT + SMIT, *p* = 0.012)
		Sum of six sprints (s), SMIT	NER	– (5.16%; *p* < 0.001)	– (7.89%; *p* < 0.001)	
		3000-m time-trial (s), HIIT	NER	– (7.35%; *p* = 0.022)	– (7.73%; *p* = 0.001)	
		3000-m time-trial (s), SMIT	NER	– (5.88%; *p* < 0.035)	– (9.50%; *p* < 0.001)	
Fisher 2017 [96] + Hoffmann 2021 [73]	40km time trial on a cycle ergometer	Time trial time (min)	F > M (*p* < 0.01)	– (1.70%; *p* < 0.01)	– (2.69%; *p* < 0.01)	NS (*p* = 0.10)
		Time trial power output (W)	M > F (*p* < 0.01)	+ (4.96%; *p* < 0.01)	+ (7.49%; *p* < 0.01)	NS (*p* = 0.14)
	Incremental cycle test *LT*_*2*_* determined using the D*_max_ *method*	Correlational analysis for Δ LT_2_ power output	N/A	NS (Δ time trial performance; r^2^ = 0.07; *p* = 0.49)	+ (related to Δ time trial performance r^2^ = 0.77; *p* = 0.004)	N/A
		Correlational analysis and Δ LT_2_ BLa, Δ absolute + relative PPO, Δ incremental time to fatigue	N/A	NS (Δ time trial performance for all)	NS (Δ time trial performance for all)	N/A
Liu 2021 [86]	YO-YO IR2 intermittent recovery test, 40-m running spaced by 10-s active rest (20-m course)	YO-YO IR2, or distance (m)	NER	+ (ES: 0.68; *p* < 0.05)	+ (ES: 0.68; *p* < 0.05)	NER
Mucci 2004 [70]	60-m running speed field test, 20-m intervals with photocells at every 20 m	Maximal running speed, *V*_max_ (m/s; max from 3 tests)	M > F (*p* < 0.001)	NS	NS	NER
		Speed at $\dot{V}$O_2max_, *v*$\dot{V}$O_2max_ (m/s)	M > F (*p* < 0.05)	+ (*p* < 0.01)	+ (*p* < 0.001)	NER
Scalzo 2014 [91]	The mean power from repeated sprints during sessions 1 and 9	Absolute mean power from 4 sprints, sessions 1 and 9 (W)	M > F (all *p* < 0.001 except 4th sprint, session 1, NS)	+ 3rd sprint session 9 vs 1 only (*p* = 0.004)	NS for all	M > F (*p* = 0.049)
		Relative mean power from sprints, sessions 1 and 9 (W/kg)	NS (*p* = 0.301)	NS	NS	NS (*p* = 0.120)
	40-km time trial on a stationary cycle ergometer	Time to cycle 40 km (min)	F > M (*p* = 0.004)	NS	NS	NS (*p* = 0.269)
Schmitz 2019 [75]	Incremental running test	Maximal speed (m/s)	NER	NER	NER	NER, pooled only
Schmitz 2020 [95]	Incremental running test	Maximal speed (km/h; baseline only)	M > F (*p* < 0.001)	NER	NER	NER, baseline only

Δ indicates change (i.e., pre-post); + denotes a significant increase; − denotes a significant decrease; *BLa* blood lactate concentration, *ES* effect size, *F* women/females, *HIIT* high-intensity interval training, *LT* lactate threshold, *LT*_*2*_ second lactate threshold, *M* men/males, *N/A* not applicable, *NER* not explicitly reported, *NS* not significant, *PPO* peak power output, *SMIT* supra-maximal interval training, *D*_*max*_ maximum-deviation, *V*_*max*_ maximal running velocity, *%*$\dot{V}$*O*_*2max*_ percent of maximal oxygen uptake, *%*$\dot{V}$*O*_*2peak*_ percent of peak oxygen uptake, *VT* ventilatory threshold, *VT*_*1*_ first ventilatory threshold, *VT*_*2*_ second ventilatory threshold, *v*$\dot{V}$*O*_*2max*_ running velocity at $\dot{V}$O_2max_, *%W*_*peak*_ percent of peak Watts

**Table 8:** Summary of outcomes and results of included studies, concurrent measures of physiological adaptation

References	Measurement	Outcome (units)	Sex, baseline	Δ M	Δ F	Interaction
Blood lactate measures						
Bornath 2022 [79]	BLa (pre- and post-) for 9th and 18th HIIT sessions *Δ* = *within session change*	BLa, Δ from rest–imm post, 9th	NS for rest	+ (ES: 0.95; *p* < 0.001)	+ (ES: 0.84; *p* < 0.001)	NER
		BLa, Δ from rest–imm post 18th	NS for imm post	+ (ES: 0.95; *p* < 0.001)	+ (ES: 0.88; *p* < 0.001)	NER
		BLa, Δ from imm–5 min post, 9th	NS for 5 min post	– (ES: 0.21; *p* = 0.048)	NS	NER
		BLa, Δ from imm–5 min post, 18th	N/A	– (ES: 0.29; *p* = 0.017)	– (ES: 0.53; *p* < 0.001)	NER
Fisher 2017 [96] + Hoffmann 2021 [73]	Incremental cycle test, earlobe blood sampling *LT*_*2*_* determined with D*_*max*_* method*	LT_2_ BLa (mmol/L)	NS	NS	NS	NS (*p* = 0.19)
Lepretre 2009 [85]	Maximal incremental cycle ergo test, capillary blood sampling	BLa (mmol 100 mL^−1^) same absolute intensity (MTP pre-)	M > F (*p* < 0.05)	– (45.03%; *p* < 0.05)	– (21.24%; *p* < 0.05)	NER
		BLa (mmol 100 mL^−1^) same relative intensity (MTP pre- vs post-)	M > F (*p* < 0.05)	NS	NS	NER
Liu 2021 [86]	Treadmill test, BLa tested at rest, 0-, 1-, 3-, 5-, 7-, and 10-min post-test	Lactate clearance rate (%)	NER	+ (ES: 2.26; *p* < 0.05)	+ (ES: 1.10; *p* < 0.05)	NER
Marterer 2020 [67]	Cycle or arm cycle ergo test, earlobe blood sampled end of each stage *LT defined as 4 mmol/L BLa*	Maximal BLa—legs (mmol/L)	NER	NS (− 6.56%; *p* = 0.083)	+ (13.33%; *p* = 0.017)	F > M (*p* = 0.003)
		Maximal BLa—arms (mmol/L)	NER	NS (*p* = 0.214)	NS (*p* = 0.920)	NS (*p* = 0.470)
Mucci 2004 [70]	Multi-stage running test, fingertip blood sampled pre- and post	Post-test BLa (mmol/L)	NS	NS	+ (*p* < 0.05)	F > M (*p* < 0.05)
Weber 2002 [74]	BLa during session 1, wk 1 [baseline] and session 3, wks 4 [mid] and 8 [post]	BLa, resting (mmol/L)	M > F	NER	NER	
		BLa, 3-min post-session (mmol/L)	M > F (*p* < 0.01)	+ (9.6 ± 3.0%; *p* < 0.05)	+ 9.6 ± 4.6%; *p* < 0.05)	NS
		BLa, 3-min post-timed cycle (mmol/L)	M > F (*p* < 0.01)	– (*p* < 0.05)	– (*p* < 0.05)	NER
Cellular and muscular adaptations						
Bagley 2016 [77] + Bagley 2021 [78]	Magnetic resonance imaging and isokinetic dynamometer	Quadriceps CSA (cm^2^)	M > F (*p* < 0.001)	+ (4.06%; *p* = 0.023)	+ (5.82%; *p* = 0.023)	NS (*p* = 0.140)
		Absolute MVC torque (Nm)	M > F (*p* < 0.001)	+ (2.76%; *p* = 0.079)	+ (5.62%; *p* = 0.079)	NS (*p* = 0.385)
		Relative MVC torque (Nm·cm^−2^)	NS (*p* = 0.248)	NS (*p* = 0.712)	NS (*p* = 0.712)	NS (*p* = 0.705)
		Quadriceps fatigue index (%)	NS (*p* = 0.282)	+ (7.67%; *p* = 0.048)	+ (15.88%; *p* = 0.048)	NS (*p* = 0.127)
Esbjörnsson Liljedahl 1996 [97]	Muscle needle biopsy	Type II fiber CSA (μm^2^)	M > F (*p* < 0.01)	NS	+ (17%; *p* < 0.05)	F > M (*p* < 0.01)
		Type II fiber (%)	NS	NS	NS	NS
		% and CSA of other fiber types	NS	NS	NS	NS
		Glycogen content	NS	NS	+ (*p* < 0.01)	F > M (*p* < 0.05)
		Total creatine content	NS	+ (*p* < 0.05)	NS	M > F (*p* < 0.05)
		Lactate dehydrogenase activity	M > F (*p* < 0.05)	+ (*p* < 0.05)	+ (*p* < 0.05)	NS
		PFK, CS or HAD	NS	NS	NS	NS
Gillen 2014 [83]	Muscle biopsy, Western blotting	CS activity	NS	+	+	NS
		β-HAD activity	F > M (*p* < 0.05)	+	NS	M > F (*p* < 0.05)
		COX IV protein content	NS	+	+	NS
		GLUT4 protein content	NS	+ (138%; *p* < 0.01)	+ (23%; *p* < 0.01)	M > F (*p* < 0.05)
Søgaard 2018 [93] + Chrøis 2020 [81]	Muscle biopsy, Western blotting	Mitochondrial respiratory capacity:				
		Coupled	NS	+ (*p* < 0.001)	NS	M > F (*p* = 0.003)
		Uncoupled	NS	+ (*p* < 0.001)	NS	M > F (*p* = 0.003)
		CS activity	NS	+ (*p* < 0.001)	+ (*p* = 0.013)	NS
		Adenosine diphosphate sensitivity	NS	– (*p* = NER)	– (*p* = NER)	NS (*p* = 0.096)
		Creatine kinase content	NS	NS	NS	NS
		Muscle glycogen content	NS	+ (*p* = 0.001)	+ (*p* = 0.001)	NS
		GLUT4	NS	+ (*p* < 0.050)	+ (*p* < 0.050)	NER
		Glycogen synthase	NS	+ (*p* = 0.001)	+ (*p* = 0.001)	NER
		Hexokinase II	NS	+ (*p* < 0.050)	+ (*p* < 0.050)	NER
		Protein expression (multiple)	NS	Varied	Varied	NS
Scalzo 2014 [91]	Muscle biopsy, D_2_O labelling (heavy water), Western blotting	Muscle mitochondrial biogenesis Muscle protein synthesis CS protein content COX IV protein content PGC1α protein content	NER M > F (*p* < 0.001) NS F > M NS	+ (*p* = NER) + (*p* = NER) + (*p* < 0.050) NS + (*p* < 0.050)	NER NER + (*p* < 0.050) NS + (*p* < 0.050)	M > F (*p* = 0.056) M > F (*p* = 0.056) NER NS NS
Central cardiorespiratory adaptations (other than $\dot{V}$O_2max_)						
Astorino 2011 [76]	Cycle ergo test, indirect calorimetry	*V*CO_2max_ (L/min)	M > F (*p* < 0.05)	+ (*p* < 0.050)	+ (*p* < 0.050)	M > F (*p* < 0.050)
		RER_max_	NS	NS	NS	NS
		HR_peak_ (bpm)	NS	NS	NS	NS
		VE_max_ (L/min)	M > F (*p* < 0.05)	NS	NS	NS
		Oxygen pulse (mL/beat)	M > F (*p* < 0.05)	+ (*p* < 0.050)	+ (*p* < 0.050)	NS (*p* = 0.30)
Bostad 2021 [80]	Cycle ergo test, indirect calorimetry Inert gas rebreathing test, approx. 20 min post $\dot{V}$O_2peak_ test *Peak cardiac index calculated as Q*_*peak*_*/body surface area*	*Q*_peak_, 6 weeks	NER	+ (*p* < 0.01)	NS	NER
		*Q*_peak_, 12 weeks	NER	+ (10%; *p* < 0.01)	NS (+ 0.6%; *p* = 0.96)	M > F (*p* < 0.01)
		SV_peak_, 6 weeks	NER	+ (*p* < 0.01)	NS	NER
		SV_peak_, 12 weeks	NER	+ (*p* < 0.01)	NS	M > F (*p* < 0.01)
		Peak cardiac index, 6 weeks	NER	+ (*p* < 0.01)	NS	NER
		Peak cardiac index, 12 weeks	NER	+ (*p* < 0.01)	NS	M > F (*p* < 0.01)
		a-$\dot{V}$O_2diff_, 6 weeks	NER	+ (*p* < 0.05)	+ (*p* < 0.05)	NER
		a-$\dot{V}$O_2diff_, 12 weeks	NER	+ (*p* < 0.01)	+ (*p* < 0.01)	NS (*p* = 0.98)
		HR_peak_ (bpm)	NER	NS	NS	NS (*p* = 0.13)
		Correlational analysis, Δ $\dot{V}$O_2peak_, *Q*_peak_, or calculated peak a-$\dot{V}$O_2diff_	N/A	NS correlation to Δ time to test completion	NS correlation to Δ time to test completion	N/A
Lepretre 2009 [85]	Maximal incremental cycle ergo test with indirect calorimetry	VE_max_ (L/min)	M > F (*p* < 0.05)	+ (7.75%; *p* < 0.05)	+ (16.93%; *p* < 0.05)	NER
Mucci 2004 [70]	Multi-stage running test, portable gas analysis and fingertip blood sampled pre- and post *EIH defined as a decrease of* > *4% in oxygen saturation during the last stage of exercise testing*	VE_max_ (L/min)	M > F (*p* < 0.01)	+ (5.77%; *p* < 0.05)	NS (+ 3.72%)	M > F (*p* < 0.001)
		HR_peak_ (bpm)	NS	– (2.47%; *p* < 0.01)	NS (− 0.63%)	NS
		VE/$\dot{V}$O_2_	NS	– (5.62%; *p* < 0.05)	NS (− 3.96%)	NS
		VE/VCO_2_	NS	– (6.25%; *p* < 0.05)	NS (− 4.73%)	NS
		Developed EIH post-HIIT	NER	*n* = 7	*n* = 2	NER
		Did not develop EIH post-HIIT	NER	*n* = 5	*n* = 8	NER
Marterer 2020 [67]	Cycle ergo test, indirect calorimetry Arm cycle test, indirect calorimetry	Oxygen pulse—legs (mL/beat)	NER	+ (11.36%; *p* < 0.001)	+ (8.94%; *p* = 0.001)	M > F (*p* = 0.008)
		HR_peak_—legs	NER	NS (*p* = 0.251)	NS (*p* = 0.234)	NS (*p* = 0.982)
		Oxygen pulse—arms (mL/beat)	NER	+ (7.39%; *p* = 0.041)	+ (9.09%; *p* = 0.004)	NS (*p* = 0.432)
		HR_peak_—arms	NER	NS (*p* = 0.833)	– (*p* = 0.013)	NS (*p* = 0.096)
		RER_max_—legs and arms	NER	NS	NS	NS
		Correlational analysis, $\dot{V}$O_2max_—legs and $\dot{V}$O_2max_—arms	N/A	NS correlation to blood volume or Hb mass	NS correlation to blood volume or Hb mass	NS
Menz 2015 [87]	Carbon monoxide rebreathing method and capillary blood samples	Total hemoglobin mass (g and g/kg)	NER	NER	NER	NS, data pooled
		Blood volume (ml/kg)	NER	NER	NER	NS, data pooled
		Plasma volume (ml/kg)	NER	NER	NER	NS, data pooled
		Maximal heart rate (bpm)	NER	NER	NER	NS, data pooled
		Maximal oxygen pulse (ml/beat)	NER	NER	NER	NS, data pooled
		RER_max_	NER	NER	NER	NS, data pooled
Weber 2002 [74]	Timed cycling test to fatigue at 120% $\dot{V}$O_2peak_, indirect calorimetry *MAOD* = *difference between AO*_*2*_* demand and AO*_*2*_* uptake*	MAOD (L), Δ pre-mid training	M > F (*p* < 0.01)	+ (14.3 ± 5.2%; *p* < 0.05)	+ (14.0 ± 3.0%; *p* < 0.01)	NS
		MAOD (L), Δ mid-post training	M > F (*p* < 0.01)	+ (6.6 ± 1.9%; *p* < 0.01)	+ (5.1 ± 2.3%; *p* < 0.05)	NS
		AO_2_ deficit (L)	M > F (*p* < 0.01)	– (*p* < 0.01)	NS	M > F (*p* = NER)
		AO_2_ uptake (L)	M > F (*p* < 0.05)	+ (*p* < 0.01)	NS	M > F (*p* = NER)
		HR_peak_ (bpm)	NS	– (*p* < 0.05)	– (*p* < 0.05)	NER

+ denotes a significant increase; − denotes a significant decrease; Δ indicates change (i.e., pre-post); *AO*_*2*_ accumulated oxygen (deficit, demand, uptake), *a-*$\dot{V}$*O*_*2diff*_ arteriovenous oxygen difference, *BLa* blood lactate concentration, *CS* citrate synthase, *CSA* cross-sectional area, *EIH* exercise-induced hypoxemia, *ES* effect size, *F* women/females, *HAD* 3-hydroxyacyl CoA dehydrogenase, *β-HAD* 3-β-hydroxyacyl CoA dehydrogenase, *Hb* hemoglobin, *HIIT* high-intensity interval training, *HR*_*peak*_ peak (maximal) heart rate, *imm* immediate, *LT*_*2*_ second lactate threshold, *D*_*max*_ maximum-deviation, *M* men/males, *MAOD* maximal accumulated oxygen deficit, *MTP* maximal tolerated power, *MVC* maximal voluntary contraction, *N/A* not applicable, *NER* not explicitly reported, *NS* not significant, *PFK* phosphofructokinase, *Q*_*peak*_ peak cardiac output, *RER*_*max*_ maximal respiratory exchange ratio, *SV*_*peak*_ peak stroke volume, *VCO*_*2*_ carbon dioxide production, *VCO*_*2max*_ maximal carbon dioxide production, *VE*_*max*_ maximal minute ventilation, $\dot{V}$*O*_*2*_ oxygen uptake

### Risk of Bias Within Studies

Quality appraisal scores for individual studies ranged from five to eight out of a maximum possible score of nine. The majority of studies (*n* = 17) were classified as good quality, indicating a low risk of bias for the current review. Eight trials were classified as fair and three trials were classified as poor. The mean inter-rater reliability for individual items of the NOS was 82.54% (± 12.17%; range 64.28–100%). The lowest inter-rater reliability scores were for the *selection of men compared to women* item and the *withdrawals and non-adherers* item. All included studies applied an equivalent intervention for males and females as indicated by question 7 on the NOS, demonstrating that prescribed exercise protocols were dose-matched between men and women within each meta-analysis. A detailed breakdown of scoring for each study can be seen in Supplementary Online Resource 5 in the ESM.

### Correlation Coefficients

Seven studies [66–72] contributed data to the imputed correlation coefficients for $\dot{V}$O_2max_, which were calculated as 0.79 (± 0.16) for women and 0.81 (± 0.10) for men. Four studies [67, 71, 73, 74] contributed data for peak power output from incremental exercise testing (PPO), which were calculated as 0.84 (± 0.07) for women and 0.81 (± 0.08) for men. Two studies [73, 75] contributed data for threshold power (power output at lactate or ventilatory threshold; power_AT_), which were calculated as 0.53 (± 0.22) for women and 0.47 (± 0.16) for men.

### Cardiorespiratory Fitness Outcomes

#### Maximal Oxygen Uptake: Study Characteristics and Primary Analysis

**Fig. 2:**
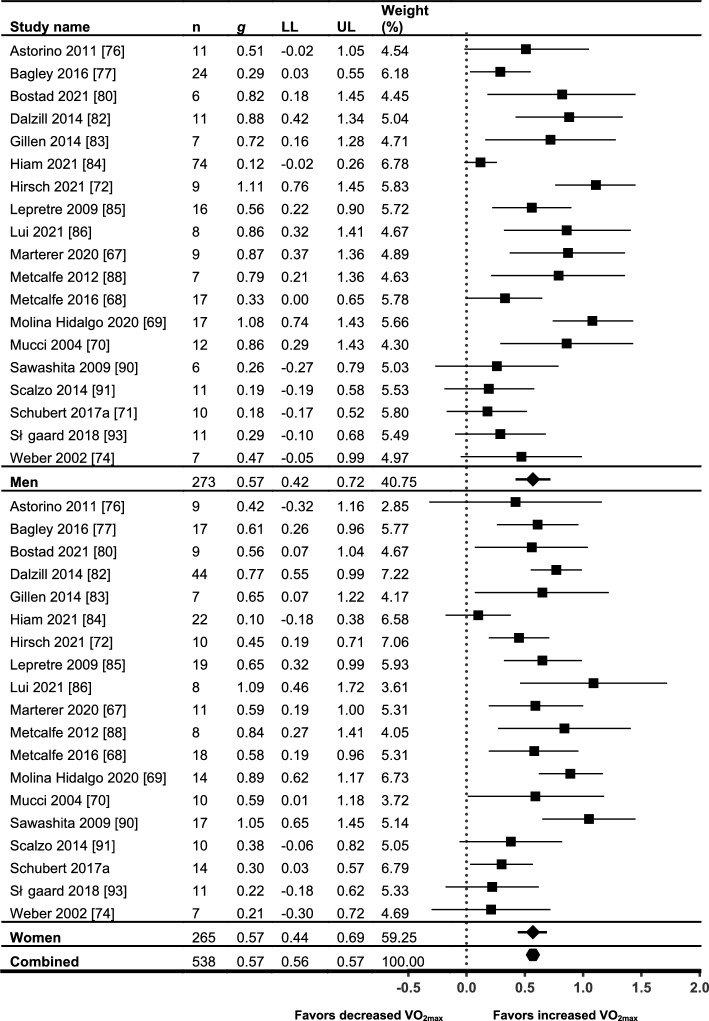
Meta-analysis of $\dot{V}$O_2max_, pre- and post-HIIT or SIT intervention. Standardized mean differences and 95% confidence intervals. *HIIT* high-intensity interval training, *SIT* sprint interval training, $\dot{V}$*O*_*2max*_ maximal oxygen uptake, *LL* confidence interval lower limit, *UL* confidence interval upper limit

Twenty-eight references from 24 individual trials [66–72, 74, 76–95] presented $\dot{V}$O_2max_ outcomes, with 19 trials presenting sufficient data for meta-analysis. A summary of all outcomes and results relating to $\dot{V}$O_2max_ is shown in Table 5. All except three of the included studies measured $\dot{V}$O_2max_ using direct calorimetry [82, 90, 95]. Of the three studies that did not measure $\dot{V}$O_2max_ using direct calorimetry, only two presented sufficient data for meta-analysis [82, 90]. Effect sizes from both studies appeared to be consistent with other studies in all analyses. Out of 32 sex × HIIT/SIT interaction analyses for $\dot{V}$O_2max_ that were reported in the primary studies, 26 were not significant. Of the six that were significant, two favored females (one study, relative and absolute $\dot{V}$O_2max_) while four favored males (four studies, all absolute $\dot{V}$O_2max_). The primary meta-analysis was undertaken to address the research question by sub-grouping study data by sex only (see Fig. 2). One study [79] demonstrated a large outlying effect size for both men and women on the initial forest plot and was excluded from all subsequent analyses (see Supplementary Online Resource 6 in the ESM). It is possible this outlying study was due to the use of an arm ergometer for measurement of $\dot{V}$O_2max_ whereas all other studies employed cycle ergometry or running protocols. This study also used an upper limb-specific HIIT training protocol using ‘battling ropes’, compared with lower limb-specific exercise modes used in all other studies. After excluding this outlying study, heterogeneity decreased from *I*^2^ = 77.26–62.14% for women and *I*^2^ = 85.97–78.80% for men. The meta-analysis demonstrated near-moderate effect sizes for increasing $\dot{V}$O_2max_ for both men (*g* = 0.57; *p* < 0.001) and women (*g* = 0.57; *p* < 0.001) with no between-group differences (*p* = 0.97). Significant levels of heterogeneity were still present for both men (*I*^2^ = 78.80%, *Q* = 84.91, *p* < 0.001) and women (*I*^2^ = 62.14%, *Q* = 47.54, *p* < 0.001).

Three studies [82, 89, 90] were categorized as poor using the NOS and AHRQ criteria, and only two had sufficient data for meta-analysis [82, 90]. Upon removing these studies, sensitivity analysis revealed small changes in effect sizes for $\dot{V}$O_2max_ in both men (*g* = 0.57; *p* < 0.001) and women (*g* = 0.52; *p* < 0.001). Similarly, only small changes in heterogeneity occurred for both men (*I*^2^ = 79.91%, *Q* = 79.64, *p* < 0.001) and women (*I*^2^ = 54.59%, *Q* = 35.23, *p* = 0.004). The impact of removing the data from these studies was considered to be small, and hence the data were retained for subsequent meta-analyses and sub-group analyses.

Initial qualitative analysis of $\dot{V}$O_2max_ outcomes revealed conflicting results regarding whether there were sex-specific differences when $\dot{V}$O_2max_ was considered in relative or absolute terms. When absolute and relative $\dot{V}$O_2max_ outcomes were retrospectively meta-analyzed separately, effect sizes were found to be similar for men and women for both outcomes, without the presence of between-group differences for either measure (Table 3; Supplementary Online Resource 7, see ESM).

The sensitivity analysis for the meta-analytical approach and for estimating raw pooled mean differences found that baseline $\dot{V}$O_2max_ was significantly higher in men compared with women for both absolute $\dot{V}$O_2max_ (between-group Δ 1.06 L·min^−1^; *p* < 0.001) and relative $\dot{V}$O_2max_ (between-group Δ 5.88 mL·kg^−1^·min^−1^; *p* < 0.001). Heterogeneity was significant for both baseline absolute and baseline relative $\dot{V}$O_2max_ (*I*^*2*^ = 70% and 60% respectively; *p* = 0.001 for both). Pre-post response to HIIT/SIT interventions measured using a raw mean difference was similar between men and women with no significant between-group differences for change in either absolute (men, Δ 0.32 L·min^−1^ vs women, Δ 0.20 L·min^−1^; *p* = 0.38) or relative $\dot{V}$O_2max_ (men, Δ 3.50 mL·kg^−1^·min^−1^ versus women, Δ 3.34 mL·kg^−1^·min^−1^; *p* = 0.88). Heterogeneity was low for the pre-post analysis of both absolute and relative $\dot{V}$O_2max_ (*I*^*2*^ = 0% for both; *p* = 0.97 and *p* = 1.0, respectively). Forest plots for this sensitivity analysis are shown in Supplementary Online Resource 8a–d (see ESM).

#### Maximal Oxygen Uptake: Sub-Groupings

**Fig. 3:**
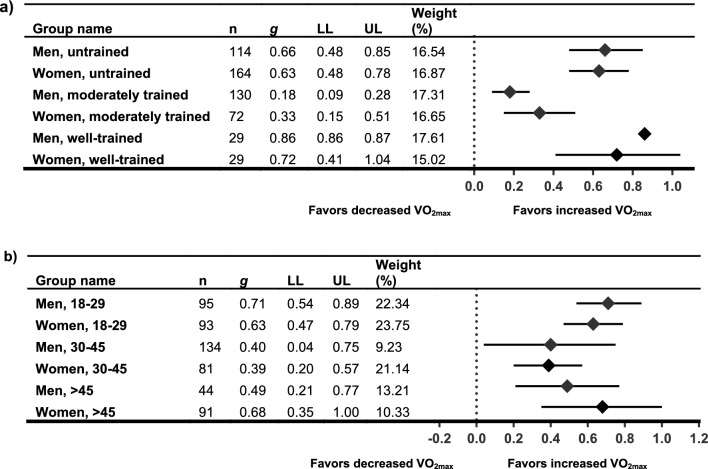
Sub-grouping of meta-analysis of $\dot{V}$O_2max_, pre- and post-HIIT or SIT intervention, **a** by baseline training status, and **b** by mean group age. Standardized mean differences and 95% confidence intervals. *HIIT* high-intensity interval training, *SIT* sprint interval training, $\dot{V}$*O*_*2max*_ maximal oxygen uptake, *LL* confidence interval lower limit, *UL* confidence interval upper limit

When $\dot{V}$O_2max_ data were stratified for training status (Fig. 3a), baseline training status accounted for significant levels of heterogeneity for moderately trained and well-trained men and women (*I*^2^ range 0.00–34.48; *p* range 1.00–0.19). Mean baseline $\dot{V}$O_2max_ in the moderately trained groups tended to sit between 35 and 48 mL·kg^−1^·min^−1^ in men, and 28 and 40 mL·kg^−1^·min^−1^ in women, whereas untrained groups and well-trained groups tended to sit below and above those ranges, respectively. The results of this meta-analysis showed significant differences overall with smaller effect sizes present in the moderately trained groups, but effect sizes for men and women were similar (Table 3).

Sub-group analysis for men and women by the mean age of the participant group (Fig. 3b) did not account for significant levels of heterogeneity in any of the sub-groups, with the exception of women under 30 years (*I*^*2*^ = 35.93; *p* = 0.12). All significant heterogeneity in the men's 30–45-year-old group was accounted to one study [72]. The exclusion of this study resulted in a substantially lowered effect size for this group (*g* = 0.18) and a significant between-group difference overall (*p* < 0.001), indicating high sensitivity and a general lack of robustness within this particular analysis. Despite the application of the age categories, participants over 45 years actually only consisted of studies with a mean age of ≥ 59 years.

**Fig. 4:**
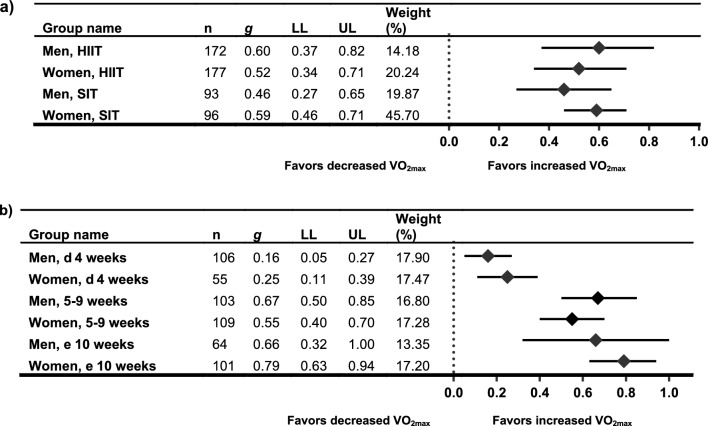
Sub-grouping of meta-analysis of $\dot{V}$O_2max_, pre- and post-HIIT or SIT intervention, **a** by intervention type, and **b** by intervention length. Standardized mean differences and 95% confidence intervals. *HIIT* high-intensity interval training, *SIT* sprint interval training, $\dot{V}$*O*_*2max*_ maximal oxygen uptake, *LL* confidence interval lower limit, *UL* confidence interval upper limit

Sub-grouping by intervention type comparing HIIT and SIT protocols demonstrated no significant between-group differences (*p* = 0.72; Fig. 4a), whereas sub-grouping by intervention length demonstrated a significant between-group difference (*p* < 0.001) with significantly smaller effect sizes present in both men and women for interventions with a duration of 4 weeks or less compared to those with a longer duration (Fig. 4b).

### Performance Outcomes

**Fig. 5:**
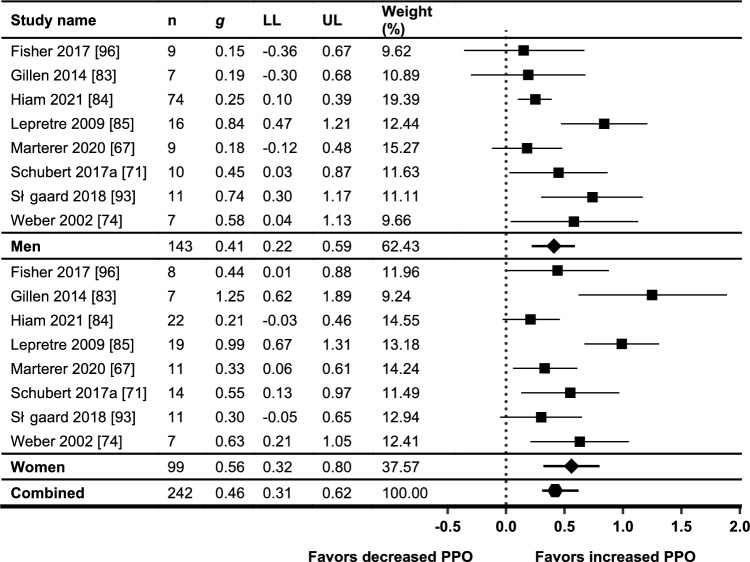
Meta-analysis of peak power out from incremental exercise testing (PPO), pre- and post-HIIT or SIT intervention. Standardized mean differences and 95% confidence intervals. *HIIT* high-intensity interval training, *SIT* sprint interval training, *LL* confidence interval lower limit, *UL* confidence interval upper limit

**Fig. 6:**
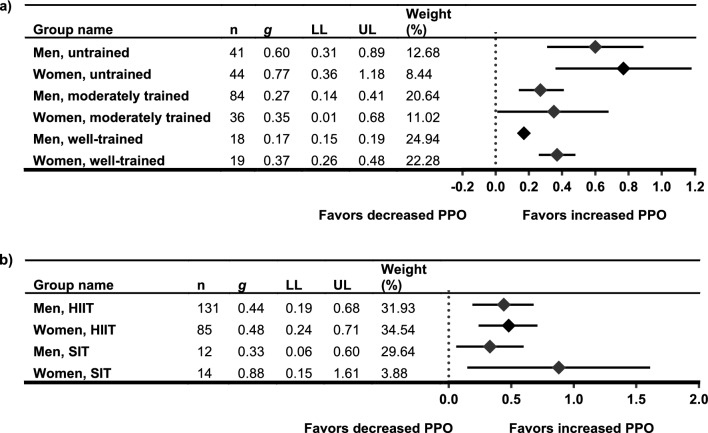
Sub-grouping of meta-analysis of peak power out from incremental exercise testing (PPO), pre- and post-HIIT or SIT intervention, **a** by baseline training status, and **b** by intervention type. Standardized mean differences and 95% confidence intervals. *HIIT* high-intensity interval training, *SIT* sprint interval training, *LL* confidence interval lower limit, *UL* confidence interval upper limit

**Fig. 7:**
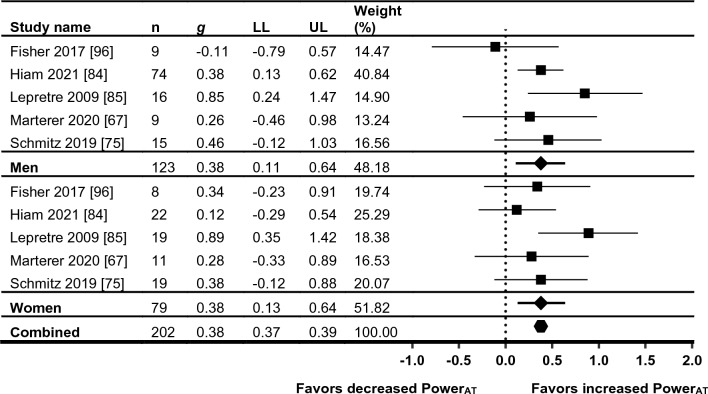
Meta-analysis of threshold power (Power_AT_), pre- and post-HIIT or SIT intervention. Standardized mean differences and 95% confidence intervals. *HIIT* high-intensity interval training, *SIT* sprint interval training, *LL* confidence interval lower limit, *UL* confidence interval upper limit

A multitude of different performance outcomes were presented in the included studies. Fourteen references from 11 individual trials presented measures of PPO [67, 71, 73, 74, 80, 81, 83–85, 89, 92–94, 96], and seven references from six individual trials presented measures of power_AT_ (*n* = 6) [67, 73, 75, 84, 85, 95, 96]. A summary of the outcomes and results for PPO and power_AT_ is outlined in Table 6. Forest plots for the meta-analyses of PPO, and PPO sub-grouped by baseline training status and intervention type, are shown in Figs. 5, 6a, b, respectively. The forest plot for power_AT_ is shown in Fig. 7. Power and $\dot{V}$O_2_-based outcomes that were not meta-analyzed included peak power output during training sessions (*n* = 2) [80, 91], threshold $\dot{V}$O_2_ (*n* = 4) [67, 72, 85, 86], Wingate outcomes (*n* = 2) [66, 76, 97], and relative and absolute power output (peak and at lactate threshold) from an arm cycle protocol (*n* = 1) [67]. Additional performance measures included time trials for running (*n* = 1) [98] and cycling (*n* = 3) [73, 80, 91], maximal speed (*n* = 2) [70, 95], 40-m sprint ability (*n* = 1) [98], repeated sprint ability (*n* = 2) [86, 98], fatigue (*n* = 4) [73, 76, 80, 93], and speed decrement (*n* = 1) [95].

#### Peak Power Output from Incremental Testing

Eight trials reporting peak power output from incremental testing were meta-analyzed [67, 74, 83–85, 92, 93, 96]. All trials tested PPO using a cycle ergometer protocol. Results demonstrated significant increases in PPO for all female and male subgroups. Women consistently demonstrated larger percent increases (6.71–13.99%) and effect sizes (*g*, range 0.35–0.77) for PPO compared to men (2.56–12.23%; *g*, range: 0.17–0.60), and the between-group difference reached the threshold for statistical significance in the sub-grouping by baseline training status, due to the larger effect size for well-trained women (*g* = 0.37) compared with well-trained men (*g* = 0.17; *p* = 0.05). Baseline training status accounted for all significant heterogeneity in PPO in moderately trained and well-trained men and women (*I*^2^, range: 0.00–54.31%; *p*, range: 0.14–0.93); however, significant levels of heterogeneity were present for the total sample and untrained sub-groups (*I*^2^, range: 54.58–79.57%; *p*, range: 0.00–0.09). Due to the smaller number of studies, PPO could not be sub-grouped by mean group age or intervention length.

#### Meta-Analysis of Threshold Power

Five trials [67, 73, 75, 84, 85] presented sufficient data to meta-analyze outcomes relating to power_AT_. No differences were demonstrated between men and women (*p* = 0.96). The percent increase in power_AT_ for men was 7.09 ± 7.17% (small effect size: *g* = 0.38; *p* < 0.01), and that for women was 8.07 ± 6.55% (small effect size: *g* = 0.38; *p* < 0.01). Some inconsistency existed in the units presented for these outcomes (e.g., work presented as Watts, W/kg, and speed in m/s) and the measures of anaerobic thresholds (lactate thresholds and ventilatory thresholds both included); however, despite this, heterogeneity was not significant, and the grouping of these outcomes appeared to be appropriate. Results demonstrated small increases in power_AT_ for men and women with low heterogeneity (men: *I*^2^ = 29.85%, *Q* = 5.70, *p* = 0.22; women: *I*^2^ = 31.26%, *Q* = 5.82, *p* = 0.21). Due to the small number of studies presenting relevant data, outcomes for power_AT_ could not be further sub-grouped.

#### Additional Performance Outcomes

A summary of results for additional performance outcomes and measures of fatigue is shown in Table 7. Most performance outcomes showed no significant differences in the magnitude of improvement between men and women. In cases where significant sex × HIIT interactions existed, these included a greater improvement in mean and maximal Wingate power output [97], repeated sprint speed decrement [95], and a 3000-m cycling time trial [98] for women compared to men. Additionally, one study demonstrated a significant correlation between the change in power output at the second lactate threshold (LT_2_) and the change in 40-km time trial performance for women (*r*^*2*^ = 0.77; *p* < 0.01) [73], while no relationships with any of the measured variables were present for men (*r*^*2*^ = 0.01–0.21; *p* all < 0.05). One study demonstrated a greater improvement in men for the mean power of the third of four repeated sprints within SIT sessions (pertaining to less power decrement over repeated sprints) [91].

### Concurrent Measures of Physiological Adaptation

Various physiological adaptions that were measured alongside other fitness and performance outcomes were reported in 15 trials [67, 70, 74, 76, 78–80, 83, 85–87, 91, 93, 96, 97]. These included maximal accumulated oxygen deficit (MAOD; *n* = 1) [74], various blood lactate measures (*n* = 8) [67, 70, 73, 74, 79, 85, 86, 95, 96], cardiac adaptations (*n* = 1) [80], mitochondrial and metabolic adaptations (*n* = 3) [81, 91, 93, 97], muscle fiber types (*n* = 1) [97], and correlational analyses for fitness or performance outcomes (*n* = 4) [67, 73, 80, 96]. A summary of the results relating to these concurrent measures of physiological adaptation is shown in Table 8.

Sex × HIIT interactions for most blood lactate and cellular or muscular measures were either not reported or not significant. Cases where significant interactions indicated greater increases in women compared to men after HIIT included maximal [67] or post-test blood lactate [70], type II muscle fiber cross-sectional area [97], and muscle glycogen content [97]. Conversely, significant interactions where men demonstrated greater increases compared to women included total muscle creatine content [97], muscle fiber β-HAD activity and GLUT4 protein content [83], coupled and uncoupled mitochondrial respiratory capacity [81], muscle mitochondrial biogenesis [91], and muscle protein synthesis [91]. All significant sex × HIIT interactions relating to central cardiorespiratory measures other than $\dot{V}$O_2max_ indicated greater changes for men compared to women following HIIT. These included significant interactions for increases in maximum carbon dioxide output [76], peak cardiac output [80], peak stroke volume [80], peak cardiac index [80], maximal minute ventilation [70], oxygen pulse [67], and accumulated oxygen uptake [74], and decreases in accumulated oxygen deficit [74].

### Assessment of Publication Bias

**Fig. 8:**
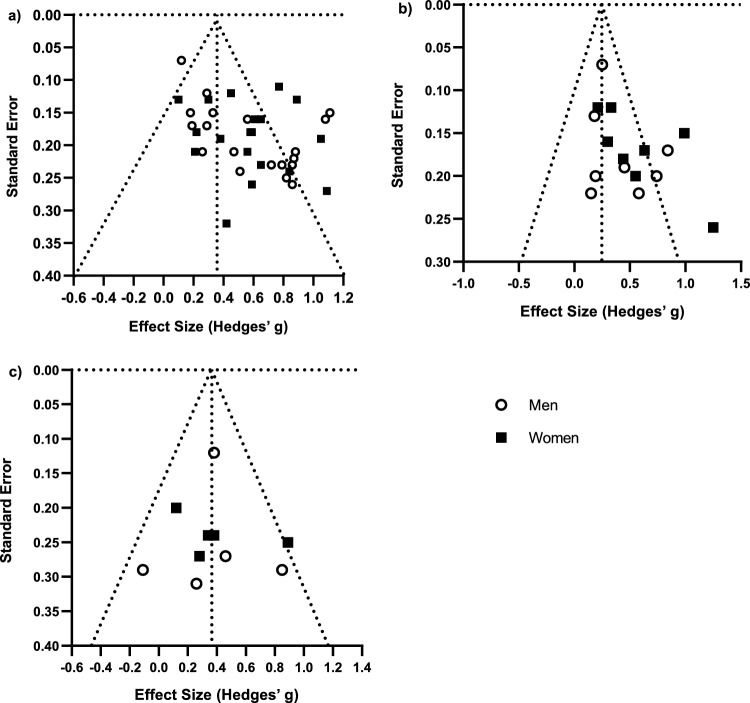
Funnel plots of the pre-post effect size (standardized mean differences; Hedges’ *g*) versus standard error for **a** maximal oxygen uptake ($\dot{V}$O_2max_), **b** peak power output from incremental exercise testing (PPO), and **c** threshold power (Power_AT_) in response to HIIT or SIT interventions. Funnels represent the 95% prediction interval for pooled, observed effect size. *HIIT* high-intensity interval training, *SIT* sprint interval training

The funnel plots for the meta-analyses of $\dot{V}$O_2max_, PPO, and power_AT_ are shown in Fig. 8a–c. Some asymmetry can be seen on the funnel plots for $\dot{V}$O_2max_ and PPO where a lack of data points can be seen at the negative effect size area of the plot. The combined male and female adjusted effect size for $\dot{V}$O_2max_ was calculated as 0.32 (95% confidence interval [CI] 0.27–0.38) compared with the observed effect size of 0.49 (95% CI 0.43–0.55). The combined adjusted effect size for PPO was calculated as 0.25 (95% CI 0.19–0.32) compared with the observed effect size of 0.41 (95% CI 0.33–0.49). Although the observed asymmetry in these funnel plots may indicate some risk of publication bias, most included studies were laboratory studies where results were based on adherence to strict protocols (see also the fidelity check in Table 2). Due to the dose–response effects of exercise load and cardiorespiratory outcomes, it is unlikely that many studies with high adherence would have produced negative overall effect sizes. Similarly, the objectives of the review necessitated this level of adherence since the aim of the review was to explore and quantify the impact of biological sex on observed physiological adaptations. As such, the presence of some asymmetry within the funnel plots may not indicate excessive publication bias within the current objectives of this review.

A visual inspection of the symmetry of the datapoint distribution for power_AT_ shows no asymmetry as an indication of notable publication bias in the pre-post effect meta-analysis. Observed and adjusted effect sizes were equivalent at 0.37 (95% CI 0.22–0.53); however, it should be noted that this meta-analysis only included data from five studies. Additionally, some reporting bias for all meta-analyzed outcomes was noted with some studies reporting a lack of sex differences in outcomes and thereby pooling male and female data. Although efforts were taken to obtain data from authors where possible, this type of reporting has resulted in some missing data from the meta-analyses. It is, however, unlikely that this missing data would have significantly affected the results for $\dot{V}$O_2max_ and power_AT_, since these strongly indicated no between-group differences in the magnitude of adaptation. The meta-analysis of PPO could have benefitted from additional data which may have influenced the final results.

## Discussion

The main finding of the current review with meta-analyses is that men and women improve fitness and performance outcomes to a similar extent following equivalent HIIT and SIT interventions. In particular, meta-analyzed outcomes for $\dot{V}$O_2max_ and power_AT_ revealed strikingly similar small to moderate increases for men and women. These findings are consistent with those of Weston and colleagues [50], who found moderate improvements in $\dot{V}$O_2max_ in active and sedentary adults in response to HIIT. Until recently, there has been insufficient research to conclude whether or not sex differences exist in fitness adaptations to HIIT interventions. Our findings expand on the work of Weston and colleagues [50], who could not come to a conclusion regarding a sex-specific response to HIIT, by demonstrating near-identical effect sizes in women and men and a lack of significant between-group differences for the primary meta-analysis of any outcome.

The sensitivity analysis that was undertaken to estimate raw mean differences found that baseline $\dot{V}$O_2max_ in men was significantly higher, the equivalent of 1.06 L·min^−1^ or 5.88 mL·kg^−1^·min^−1^, compared with women, as expected. The pre-post analysis using a raw mean difference indicated significant overall improvements of approximately 0.23 L·min^−1^ and 3.40 mL·kg^−1^·min^−1^, without the presence of significant between-group differences for men and women. This analysis indicates that the general results were not altered by the use of a standardized mean difference designed for dependent data and provides more practical estimates of effect. The results of this analysis should be taken with some caution, however, since the meta-analytical approach assumes independent data. In particular, the low levels of heterogeneity in the pre-post analyses appear to be underestimated.

While larger effect sizes for PPO were demonstrated for women compared with men across all sub-groupings, this difference only reached the threshold of significance in participants who were well trained at baseline. Despite the presence of this apparent difference, it must be noted that the well-trained male and female sub-groups only consisted of two studies and had a small sample size of only 18 men and 19 women. Baseline training status accounted for all significant heterogeneity in $\dot{V}$O_2max_ and PPO outcomes in moderately trained and well-trained men and women. While decreases in the variability of outcomes for participants who were trained at baseline could make sense from a physiological standpoint as fitness and performance outcomes move closer to a theoretical physiological ceiling, it is unclear from the current analysis what factors contributed to the variability of outcomes for untrained participants.

A significant between-group difference was present overall for $\dot{V}$O_2max_ when sub-grouped by baseline training status, with smaller effect sizes present for the moderately trained groups. This likely reflected some confounding from shorter intervention lengths in the moderately trained groups. All except one study within the moderately trained groups had an intervention length ranging between 2 and 4 weeks, while studies in the well-trained category had a range of 6–8 weeks. Consistent with this, the sub-grouping by intervention length revealed significant between-group differences with smaller effect sizes seen for interventions of ≤ 4 weeks in duration for both sexes. While the female data demonstrated a gradual increase in effect size with longer interventions, the male data demonstrated similar effect sizes but greater variability with all sub-groups longer than 4 weeks in duration. Interestingly, these findings are consistent with the study by Hirsch et al. [72], who highlighted potential differences in the rate of adaptation between men and women, with significant changes in $\dot{V}$O_2max_ occurring during the first 4 weeks for the men in their study, while the significant changes in women occurred during the second 4 weeks.

Conversely, the sub-group analyses for mean group age and intervention type (HIIT versus SIT) did not significantly influence the heterogeneity in $\dot{V}$O_2max_ or PPO outcomes and revealed no significant between-group differences. Furthermore, the observed sensitivity of the mean age sub-group analysis indicated fundamental issues with the robustness of this grouping, and as such these results should be interpreted with caution. Overall, it appears that baseline training status and intervention length may be important factors influencing the variability of outcomes in response to HIIT and SIT interventions for both sexes, rather than age or intervention type.

Except for conflicting evidence regarding fatigue and speed/power decrement, the qualitative analysis of results of the additional performance outcomes (outlined in Table 7) demonstrated a similar lack of differences between men and women regarding the magnitude of change for most performance outcomes. Generally, the included studies supported current knowledge that women are less fatigable than men [66, 73, 76, 91, 95, 96] prior to HIIT. While some of the studies included in this review indicated that men may be able to adapt this to a greater extent than women through HIIT [91, 93], some studies found significant improvements in fatigability or power output through repeated efforts in women only [95, 97], while still other studies found no sex differences at all regarding the change in fatigability [66, 73, 76, 96].

Despite the notable lack of sex differences in the magnitude of adaptation of fitness and performance outcomes in response to HIIT, some of the findings outlined in Tables 7 and 8 indicated potential differences regarding the underlying mechanisms contributing to these improvements. Most notably, all significant sex × HIIT interactions reported in the primary studies that related to central cardiorespiratory adaptations favored men, with women generally demonstrating smaller, and often non-significant changes. Examples of this included the observed increases in peak cardiac output, peak stroke volume, and peak cardiac index in men only after SIT, as demonstrated by Bostad et al. [80]; the significant sex × HIIT interaction for oxygen pulse (the amount of oxygen ejected from the ventricles with each cardiac contraction) with a greater increase in men compared with women as demonstrated by Marterer and colleagues [67]; the increases in accumulated oxygen uptake and decreases in accumulated oxygen deficit after HIIT seen only in men, as demonstrated by Weber and Schneider [74]; and finally, the increases in minute ventilation and decreases in maximum heart rate and the ventilatory equivalents for oxygen and carbon dioxide in men only, as demonstrated by Mucci and colleagues [70]. These findings, together with the increase in coupled and uncoupled mitochondrial respiratory capacity that was demonstrated in men only, as outlined by Chrøis et al. [81], indicate that improvements in oxygen delivery and uptake in men may play a greater role in achieving fitness and performance adaptations after HIIT compared with women. While adaptations to high-intensity exercise via improvements in oxygen delivery and uptake may seem counter-intuitive on initial contemplation, previous studies have indicated that the greater oxygen availability during exercise in women provides an advantage with regard to the fatigability of muscular contractions, even at maximal intensities [43, 99]. Consistent with this, it appears that the sex differences in fatigability can be eliminated under ischemic conditions [100]. As such, it is conceivable that such adaptations may contribute to improvements in maximal fitness and performance outcomes. Similar findings have also been reported in a previous review of responses to endurance training, where increases in left ventricular end-diastolic volume and stroke volume increased to a greater extent in men compared with women [101]. Although this meta-analysis reported outcomes using mean differences, thereby reporting absolute changes in these outcomes, the results reported in the primary studies included in the current review indicate that these differences may also exist in these changes when considered relative to baseline.

In contrast, there was a relative lack of evidence surrounding the mechanisms of adaptation that account for the equivalent improvements in fitness and performance outcomes in women. Interestingly, the study by Hoffmann and colleagues [73] reported a significant correlation where a change in threshold power (LT_2_) accounted for 77% of the performance improvement in the 40 km cycling time trial for women. This same result was not observed for men, nor were there any significant correlations between change in time trial performance and measures of heart rate or blood lactate at LT_2_, absolute or relative peak power output, or incremental time to fatigue. Another study in the current review [97] found a significant increase in mean power during repeated Wingate tests and greater increases in the cross-sectional area of type IIb muscle fibers in women only in response to four weeks of SIT. Although these are the findings of only two studies, which could have been influenced by exercise mode since both used cycle-based testing and training protocols, some additional information can be gathered from outcomes that have been presented in other contexts. Spina and colleagues [102] compared mechanisms of adaptation in older men and women who participated in 9–12 months of moderate to vigorous uphill walking and running-based endurance training and found that 66% of the $\dot{V}$O_2max_ adaption in older men was accounted for by a 15% increase in stroke volume in combination with a 7% increase in arteriovenous oxygen difference at maximal exercise. In contrast, this study found no change in stroke volume in older women and the whole change in $\dot{V}$O_2max_ could only be contributed to an increase in peripheral oxygen extraction. In another example, similar to the relationship noted by Hoffmann and colleagues [73], one cross-sectional study [103] found that 60% of the variability in 10-km performance for highly trained female runners aged 23–47 years was explained by running velocity at the lactate threshold. The authors noted, however, that this relationship was age-dependent, with $\dot{V}$O_2max_ explaining 74% of the variability in performance in women aged 37–56 years. The potential differences highlighted here alongside the strikingly similar effect sizes seen for $\dot{V}$O_2max_ in the current meta-analysis suggest some potential sex differences in the adaptive responses to HIIT/SIT that may warrant further investigation.

Despite highlighting potential differences in the adaptative responses to HIIT and SIT in men and women, these appear to be only different means to the same end. Improvements in maximal cardiorespiratory fitness along with many other performance measures outlined in the results of the current review were found to occur to a similar magnitude in men and women. In contrast to this, a previous meta-analysis by Diaz-Canestro and Montero [52] reported greater increases in absolute and relative $\dot{V}$O_2max_ in men compared with women in response to moderate-intensity endurance training. This discrepancy between the results of these two meta-analyses may be influenced by the differing interventions (endurance training vs HIIT/SIT) or the methodological differences between the two meta-analyses. The earlier review by Diaz-Canestro and Montero [52] presented absolute change in $\dot{V}$O_2max_ (mL·kg^−1^·min^−1^ and mL·min^−1^) using raw mean differences, whereas the current review primarily focused on changes relative to baseline reflected as standardized effect sizes, percentage change, and sex × HIIT interactions (see also the comment by Senefeld and colleagues [104]). Despite these differences in overall approach, the sensitivity analysis using a raw mean difference in the current review persisted to indicate that there were no significant differences between men and women for either absolute or relative $\dot{V}$O_2max_ in response to HIIT/SIT interventions. Overall, these findings seem to indicate that sex differences in $\dot{V}$O_2max_ response may be protocol dependent, and could warrant further investigation.

## Limitations

The current review has a number of limitations. Firstly, in order to minimize confounding from different exercise protocols (exercise dose) across studies, only studies that presented both male and female data were included in the current review. While this ensures that exercise dose is normalized between male and female sub-groups and provides relatively similar numbers of men and women within each analysis, the majority of the included studies were small, which limits the number of participants within the meta-analyses overall. Since studies with small sample sizes tend to be associated with larger effect sizes and greater error, some of the effect sizes seen here have the potential to be overestimated or unduly influenced by a small number of participants. Despite this, the focus of the current review was to identify differences between the relative change in outcomes for men and women rather than quantifying effect sizes. The general effects of HIIT, particularly with relation to $\dot{V}$O_2max_, have been demonstrated for mixed male and female groups in previous meta-analyses [4, 9, 50], and the current analysis strongly indicates that sex differences in the relative change in these outcomes are minimal.

Another potential limitation of the current meta-analysis could be that while prescribed training doses were matched between men and women in all studies, there was an inability to properly assess the actual dose received in many studies and whether this was consistent with the prescribed dose (dose delivered). Many studies provided only basic details regarding compliance to prescribed exercise, with only a few reporting that this remained consistent between men and women. Despite this, many of the included studies appeared to have been tightly controlled interventions, in which case the differences between the prescribed and actual training doses are unlikely to have been substantially different.

In addition to the small pooled sample size and challenges associated with assessing the fidelity of the interventions, the use of pre-post meta-analysis techniques and multiple effect sizes from individual studies in the same analysis (such as the matched male and female sub-group data) have been widely used, but also widely debated in the literature [105–107]. While the use of dependent pre-post data has been largely accounted for with the use of correlation coefficients in the meta-analyses, sufficient data was not available to calculate correlation coefficients for all studies, therefore many of these were imputed. Furthermore, while the design of the current review ensures that prescribed exercise dose is matched between male and female groups, the wide range of intervention lengths and exercise protocols included in the literature makes it difficult to precisely examine the influence of different protocols on these outcomes. Overall, the approaches used within the current review with meta-analyses aimed to minimize statistical and methodological errors as much as possible in the face of the unique set of challenges associated with the research question; however, the results should be considered within the constraints of the limitations that are outlined here.

Finally, while the current review provides some insight into generalized similarities and differences between men and women regarding physiological adaptations to HIIT interventions, evaluation of the influence of hormonal status on outcomes was outside of the scope of this review. While the influence of hormonal fluctuations in women may be somewhat offset by the current focus on adaptation over several sessions, generally spanning weeks or months, these concepts appeared to be largely overlooked in the included studies and should be a focus of future primary and secondary research. Despite the limitations outlined here, the findings from the current review will be critical in order to fill the research gaps and to promote better optimization of exercise prescription and health for both women and men.

## Conclusions

The current review with meta-analyses aimed to clarify sex differences in the adaptations of fitness and performance outcomes in response to HIIT and SIT interventions. The main findings of this review indicated that the magnitude of change in $\dot{V}$O_2max_ and power_AT_ in response to HIIT interventions is similar for men and women. While a borderline significant sex difference was found for PPO in well-trained men and women, the sub-groups consisted of small sample sizes and therefore should be interpreted with caution. Additionally, qualitative analysis of performance outcomes and concurrent measures of physiological adaptation indicated potential differences in the underlying mechanisms of adaptation for men and women. Lastly, it appears that baseline training status and intervention length may play a role in influencing the variability of $\dot{V}$O_2max_ and PPO outcomes in both sexes, including significantly smaller effect sizes for interventions with a duration of 4 weeks or less.

### Supplementary Information

Below is the link to the electronic supplementary material.

Supplementary file1 (XLSX 158 KB)

Supplementary file2 (PDF 1577 KB)

## Declarations

### Data availability

The extracted data that was used for the current manuscript is available in Supplementary Online Resource 1 in the ESM.
