# Supplementary material for: Cardiorespiratory Fitness and Performance Adaptations to High-Intensity Interval Training: Are There Differences Between Men and Women? A Systematic Review with Meta-Analyses
Source: Sports Med. 2023 Sep 7;54(1):127–67. doi: 10.1007/s40279-023-01914-0 (PMC10799129; doi:10.1007/s40279-023-01914-0)

Supplementary online resource 2 Full search strategy as undertaken in MEDLINE

S8	S1 AND S7
S7	S4 OR S6
S6	S2 AND S3 AND S5
S5	interval OR intermittent
S4	HIIT OR HIT OR HIIE
S3	high intensity OR high-intensity OR vigorous OR maximal OR sprint
S2	((ZW "exercise") or (ZW "exercise & training") or (ZW "exercise (ex)") or (ZW "exercise (mesh d015444)") or (ZW "exercise / physical activity") or (ZW "exercise [mesh]") or (ZW "exercise activity") or (ZW "exercise adaptation") or (ZW "exercise adaptations")) OR workout or workouts OR intervention
S1	sex OR gender OR (females or women or female or woman) AND (males or men or male or man)

Supplementary online resource 3 Newcastle - Ottawa quality assessment scale for case control studies [1], adapted for assessing bias for sex differences in outcomes to HIIT

Note: A study can be awarded a maximum of one star for each numbered item within the Selection and Exposure categories. A maximum of two stars can be given for Comparability.

Scale item	Detailed explanation	Star rating
**Selection**		
**1) Are the outcome measures adequate?** ***('outcome measures' vs 'case definition')***		
a) yes, with independent validation	*The outcome measures used have good validity; objective/gold standard where possible; preferably researcher blinded if the study is controlled; likely to have accurately measured the outcome of interest.*	★
b) yes, eg record linkage or based on self-reports	*Potentially valid outcome measures or unclear; estimated (not measured directly) or only somewhat likely to have accurately measured the outcome of interest*	No star
c) no description	*No description or inadequate measures used*	No star
**2) Representativeness of the cases**		
a) consecutive or obviously representative series of cases	*Low risk of selection bias such as a large sample size, not self-selected into the intervention or whole target groups included; randomised controlled trial design*	★
b) potential for selection biases	*Moderate risk of selection bias (most likely); participants self-selected into research study; non-controlled study design*	No star
**3) Selection of controls** ***(male group, in comparison to females)***		
a) community controls	*Male participants recruited from the same community or population as female participants. Same recruitment strategies used.*	★
b) hospital controls	*Male participants recruited from a different community or population as female participants or different recruitment strategies used.*	No star
c) no description	*No description of the community or population that male or female participants were recruited.*	No star
**4) Definition of controls** ***– same inclusion criteria used for selection of male and female participants (i.e. baseline fitness (relative) or training level, pathology or risk factors between groups)***		
a) no history of disease (endpoint)	*Same inclusion criteria used and no significant differences in baseline fitness (relative) or training level, pathology or risk factors between groups are present*	★
b) no description of source	*Differing inclusion criteria used or significant differences in baseline fitness (relative) or training level, pathology or risk factors between groups are present*	No star
**Comparability (sex groups)**		
**1) Comparability of** *males and females* **on the basis of the design or analysis**		
a) study controls for *age*	*Male and female groups are of a similar mean age at baseline*	★
b) study controls (accounts) for *menstrual cycle*	*Study design or analysis accounts for menstrual cycle (i.e. female participants are tested at the same time during their cycle or other means of accounting for the cycle are used)*	★
**Exposure (intervention)**		
**1) Ascertainment of exposure** ***- Description of intervention – has the intervention been applied equitably for both genders? (i.e. for HIIT example, intensities used were relative to individual maximal performance)***		
a) secure record (eg surgical records)	*Participants completed an equitable intervention with good adherence and researcher validation (lab-based or supervised exercise sessions with non-adherers excluded)*	★
b) structured interview where blind to case/control status	*Participants completed an equitable intervention with good adherence but self-reported adherence (non-supervised exercise sessions with non-adherers excluded)*	★
c) interview not blinded to case/control status -	*Equitable intervention with non-adherers NOT excluded*	No star
d) written self-report or medical record only	*Non-equitable intervention – intervention is not relatively similar for males and females*	No star

e) no description	*Inadequate description of intervention*	No star
**2) Same method of ascertainment for *males and females***		
a) yes	*Adherence was tracked using the same method for both male and female participants.*	★
b) no	*Adherence was tracked using the differing methods for both male and female participants.*	No star
**3) Non-response rate *(withdrawals from the intervention and non-adherers)***		
a) same rate for *males and females*	*Withdrawal and non-adherence rates were similar for male and female participants*	★
b) non respondents described	*Withdrawal and non-adherence rates were not similar for male and female participants, but differences described in limitations or accounted for in analysis*	No star
c) rate different and no designation	*Withdrawal and non-adherence rates were not similar for male and female participants, and differences were NOT described in limitations or accounted for in analysis*	No star

Maximum possible score being ten stars with the higher the number of stars the high level of quality (lower risk of bias) for each individual study.

References

[1] Wells G, Shea B, O'Connell D, Robertson J, Peterson J, Losos M, et al. The Newcastle-Ottawa Scale (NOS) for assessing the quality of nonrandomized studies in meta- analysis.

Supplementary online resource 4 PRISMA 2020 checklist

Section and Topic	Item #	Checklist item	Location where item is reported
**TITLE**			
Title	1	Identify the report as a systematic review.	P1
**ABSTRACT**			
Abstract	2	See the PRISMA 2020 for Abstracts checklist.	P3-4
**INTRODUCTION**			
Rationale	3	Describe the rationale for the review in the context of existing knowledge.	P5-7
Objectives	4	Provide an explicit statement of the objective(s) or question(s) the review addresses.	P7
**METHODS**			
Eligibility criteria	5	Specify the inclusion and exclusion criteria for the review and how studies were grouped for the syntheses.	P8<br>Section 2.2
Information sources	6	Specify all databases, registers, websites, organisations, reference lists and other sources searched or consulted to identify studies. Specify the date when each source was last searched or consulted.	P7<br>Section 2.2
Search strategy	7	Present the full search strategies for all databases, registers and websites, including any filters and limits used.	P7<br>Section 2.2<br>Supplementary Online Resource 2
Selection process	8	Specify the methods used to decide whether a study met the inclusion criteria of the review, including how many reviewers screened each record and each report retrieved, whether they worked independently, and if applicable, details of automation tools used in the process.	P8<br>Section 2.2
Data collection process	9	Specify the methods used to collect data from reports, including how many reviewers collected data from each report, whether they worked independently, any processes for obtaining or confirming data from study investigators, and if applicable, details of automation tools used in the process.	P9<br>Section 2.4
Data items	10a	List and define all outcomes for which data were sought. Specify whether all results that were compatible with each outcome domain in each study were sought (e.g. for all measures, time points, analyses), and if not, the methods used to decide which results to collect.	P9-10<br>Section 2.4
	10b	List and define all other variables for which data were sought (e.g. participant and intervention characteristics, funding sources). Describe any assumptions made about any missing or unclear information.	P9-10<br>Section 2.4
Study risk of bias assessment	11	Specify the methods used to assess risk of bias in the included studies, including details of the tool(s) used, how many reviewers assessed each study and whether they worked independently, and if applicable, details of automation tools used in the process.	P9<br>Section 2.3<br>Supplementary Online Resource 3
Effect measures	12	Specify for each outcome the effect measure(s) (e.g. risk ratio, mean difference) used in the synthesis or presentation of results.	P9-10<br>Section 2.4
Synthesis	13a	Describe the processes used to decide which studies were eligible for each synthesis (e.g. tabulating the study intervention	P9-10

Section and Topic	Item #	Checklist item	Location where item is reported
methods		characteristics and comparing against the planned groups for each synthesis (item #5)).	Section 2.4
	13b	Describe any methods required to prepare the data for presentation or synthesis, such as handling of missing summary statistics, or data conversions.	P9-10<br>Section 2.4
	13c	Describe any methods used to tabulate or visually display results of individual studies and syntheses.	P9-11<br>Sections 2.4-2.6
	13d	Describe any methods used to synthesize results and provide a rationale for the choice(s). If meta-analysis was performed, describe the model(s), method(s) to identify the presence and extent of statistical heterogeneity, and software package(s) used.	P9-10<br>Sections 2.4-2.6
	13e	Describe any methods used to explore possible causes of heterogeneity among study results (e.g. subgroup analysis, meta-regression).	P10<br>Section 2.5
	13f	Describe any sensitivity analyses conducted to assess robustness of the synthesized results.	P10<br>Section 2.5
Reporting bias assessment	14	Describe any methods used to assess risk of bias due to missing results in a synthesis (arising from reporting biases).	P11<br>Section 2.7
Certainty assessment	15	Describe any methods used to assess certainty (or confidence) in the body of evidence for an outcome.	P10-P11<br>Sections 2.5-2.7
**RESULTS**			
Study selection	16a	Describe the results of the search and selection process, from the number of records identified in the search to the number of studies included in the review, ideally using a flow diagram.	P11<br>Section 3.1<br>PRISMA diagram<br>Figure 1
	16b	Cite studies that might appear to meet the inclusion criteria, but which were excluded, and explain why they were excluded.	P11-12<br>Sections 3.1 and 3.4.1<br>Supplementary Online Resource 6
Study characteristics	17	Cite each included study and present its characteristics.	Table 1<br>Table 2
Risk of bias in studies	18	Present assessments of risk of bias for each included study.	Supplementary Online Resource 5
Results of individual studies	19	For all outcomes, present, for each study: (a) summary statistics for each group (where appropriate) and (b) an effect estimate and its precision (e.g. confidence/credible interval), ideally using structured tables or plots.	P12-16<br>Sections 3.4-3.6<br>Tables 5-8
Results of syntheses	20a	For each synthesis, briefly summarise the characteristics and risk of bias among contributing studies.	P12<br>3.2
	20b	Present results of all statistical syntheses conducted. If meta-analysis was done, present for each the summary estimate and its precision (e.g. confidence/credible interval) and measures of statistical heterogeneity. If comparing groups, describe the direction of the effect.	Figures 2-7

Section and Topic	Item #	Checklist item	Location where item is reported
			Tables 3-4<br>Supplementary Online Resource 7
	20c	Present results of all investigations of possible causes of heterogeneity among study results.	P12-15<br>Sections 3.4-3.6<br>Tables 3-4
	20d	Present results of all sensitivity analyses conducted to assess the robustness of the synthesized results.	P12-15<br>Sections 3.4-3.6
Reporting biases	21	Present assessments of risk of bias due to missing results (arising from reporting biases) for each synthesis assessed.	P16-17<br>Section 3.7<br>Figure 8a, b, and c
Certainty of evidence	22	Present assessments of certainty (or confidence) in the body of evidence for each outcome assessed.	P16-17<br>Section 3.7<br>Figure 8a, b, and c
**DISCUSSION**			
Discussion	23a	Provide a general interpretation of the results in the context of other evidence.	P17-21<br>Section 4.0
	23b	Discuss any limitations of the evidence included in the review.	P20-21<br>Section 4.0
	23c	Discuss any limitations of the review processes used.	P20-21<br>Section 4.0
	23d	Discuss implications of the results for practice, policy, and future research.	P17-21<br>Section 4.0
**OTHER INFORMATION**			
Registration and protocol	24a	Provide registration information for the review, including register name and registration number, or state that the review was not registered.	P7<br>Section 2.2
	24b	Indicate where the review protocol can be accessed, or state that a protocol was not prepared.	P7<br>Section 2.2
	24c	Describe and explain any amendments to information provided at registration or in the protocol.	P7-11<br>Sections 2.2-2.5
Support	25	Describe sources of financial or non-financial support for the review, and the role of the funders or sponsors in the review.	P2
Competing interests	26	Declare any competing interests of review authors.	P2
Availability of data, code and other materials	27	Report which of the following are publicly available and where they can be found: template data collection forms; data extracted from included studies; data used for all analyses; analytic code; any other materials used in the review.	Tables 1-8<br>Figures 1-8<br>Supplementary Online Resource 1-

Section and Topic	Item #	Checklist item	Location where item is reported
			7

Supplementary online resource 5 Breakdown of scoring for individual items of the Newcastle-Ottawa Scale for included studies and categorisation according to the Agency for Healthcare Research and Quality (AHRQ) thresholds*

**Domain**	**Selection**				**Comparability (sex groups)**		**Exposure (intervention)**			**Total score**	**Category**
**Scoring item**	1) Are the outcome measures adequate?	2) Representativeness of the cases	3) Selection of males compared to females	4) Definition of comparison group	5) Comparability of males and females on the basis of the design or analysis		6) Ascertainment of exposure - Description of intervention	7) Same method for males and females	8) Withdrawals and non-adherers		
**Reference**					a) study controls for age	b) study controls (accounts) for menstrual cycle					
Astorino 2011 [76] + Astorino 2012 [66]	✓		✓	✓	✓		✓	✓	✓	7	Good
Bagley 2016 [77] + Bagley 2021 [78]	✓			✓	✓		✓	✓	✓	6	Fair
Bornath 2022 [79]	✓		✓	✓	✓		✓	✓	✓	7	Good
Bostad 2021 [80]	✓		✓	✓	✓		✓	✓	✓	7	Good
Chrøis 2020 [81] + Søgaard 2018 [93]	✓		✓	✓	✓		✓	✓	✓	7	Good
Cicioni-Kolsky 2013 [98]	✓	✓	✓	✓	✓		✓	✓	✓	8	Good
Dalzill 2014 [82]		✓	✓	✓			✓	✓	✓	6	Poor
Esbjörnsson Liljedahl 1996 [97]	✓		✓	✓	✓		✓	✓	✓	7	Good
Fisher 2017 [96] + Hoffmann 2021 [73]	✓		✓	✓	✓	✓	✓	✓	✓	8	Good
Gillen 2014 [83]	✓		✓	✓	✓		✓	✓	✓	7	Good
Hiam 2021 [84]	✓		✓	✓	✓		✓	✓	✓	7	Good
Hirsch 2021 [72]	✓			✓	✓	✓	✓	✓	✓	7	Fair
Lepretre 2009 [85]	✓			✓	✓	✓	✓	✓	✓	7	Fair
Liu 2021 [86]	✓	✓	✓	✓	✓		✓	✓	✓	8	Good
Marterer 2020 [67]	✓		✓		✓		✓	✓	✓	6	Fair
Menz 2015 [87]	✓		✓	✓	✓		✓	✓	✓	7	Good
Metcalfe 2012 [88]	✓		✓	✓	✓	✓	✓	✓	✓	8	Good
Metcalfe 2016 [68]	✓		✓	✓	✓	✓	✓	✓	✓	8	Good
Molina-Hidalgo 2020 [69]	✓	✓	✓	✓	✓		✓	✓	✓	8	Good
Mucci 2004 [70]	✓		✓	✓	✓	✓	✓	✓	✓	8	Good
Phillips 2017 [89]	✓	✓	✓				✓	✓	✓	6	Poor
Sawashita 2009 [90]				✓	✓		✓	✓	✓	5	Poor
Scalzo 2014 [91]	✓		✓	✓	✓		✓	✓	✓	7	Good

Schmitz 2020 [95]			✓	✓	✓		✓	✓		5	Fair
Schmitz 2019 [75]	✓		✓		✓		✓	✓	✓	6	Fair
Schubert 2017a [71] + Schubert 2017b [92]	✓		✓			✓	✓	✓	✓	6	Fair
Støren 2017 [94]	✓		✓		✓		✓	✓	✓	6	Fair
Weber 2002 [74]	✓		✓	✓	✓	✓	✓	✓	✓	8	Good
**IRR (% similarity)**	92.59	74.07	66.67	81.48	85.19	92.59	88.89	100	66.67		
**IRR Mean (±SD) of individual items**									83.13 (±11.87)		
									**Total score, mean (±SD)**	6.89 (0.93)	

*Domain scores were used to categorize studies into good, fair, and poor quality using the following thresholds outlined by the Agency for Healthcare Research and Quality (AHRQ).

- Good quality: must have scored three or four stars in the selection domain, as well as one or two stars in the comparability domain, and two or three stars in the exposure (intervention) domain.
- Fair quality: two stars in the selection domain, one or two stars in the comparability domain, and two or three stars in the exposure (intervention) domain.
- Poor-quality studies if they scored zero or one star in the selection domain, zero stars in the comparability domain, or zero or one star in the exposure (intervention) domain.

Supplementary online resource 6 Forest plot showing Bornath 2022 study as an outlier

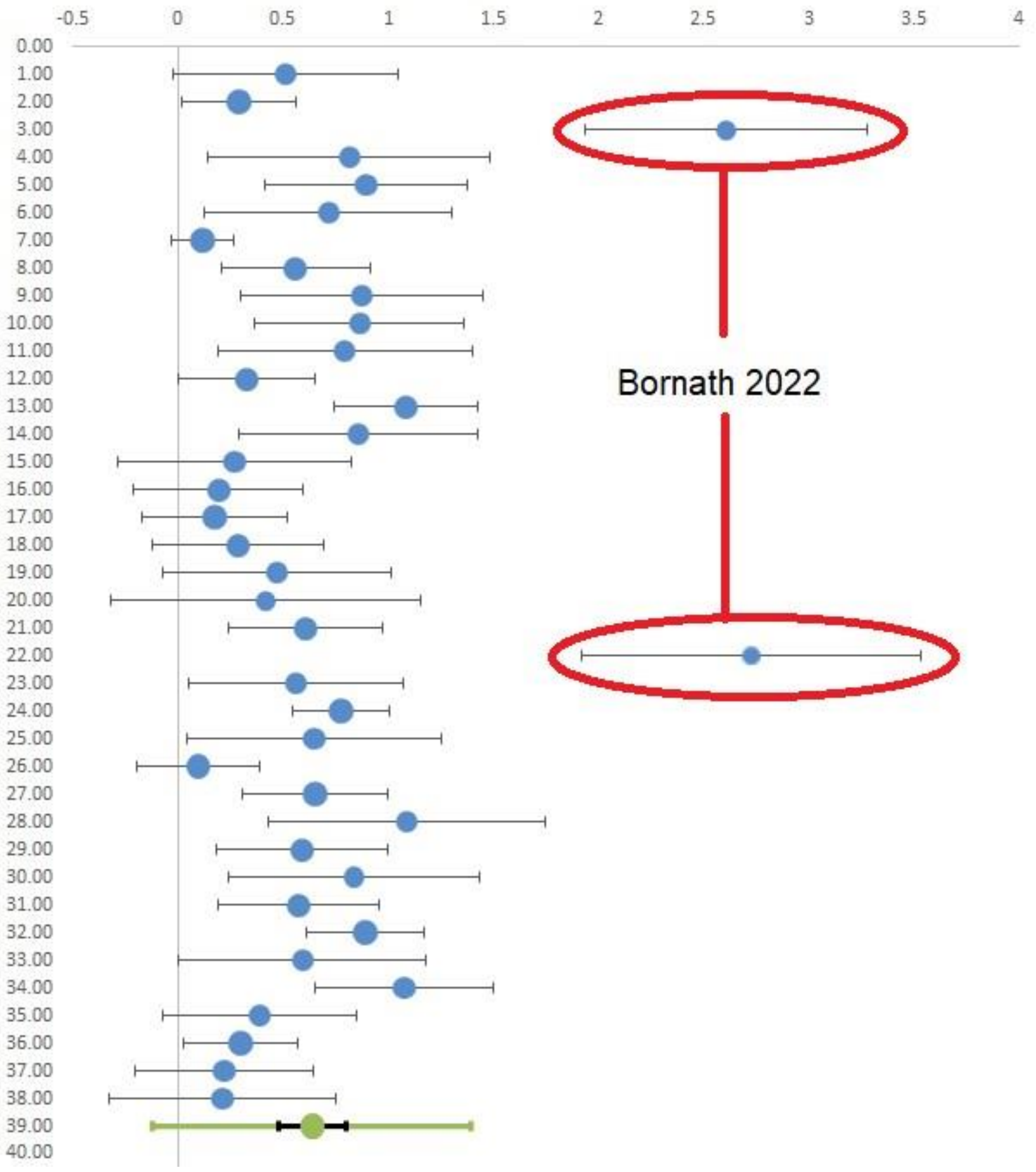

Supplementary online resource 7 Forest plot of pooled absolute and relative VO_{2max} outcomes

Group name	n	*g*	LL	UL	Weight (%)
Men, absolute	91	0.51	0.32	0.70	68.39
Women, absolute	87	0.56	0.28	0.83	31.61
Combined absolute	178	0.53	0.48	0.57	100.00
Men, relative	249	0.55	0.38	0.71	40.24
Women, relative	205	0.57	0.43	0.71	59.76
Combined relative	454	0.56	0.54	0.58	100.00

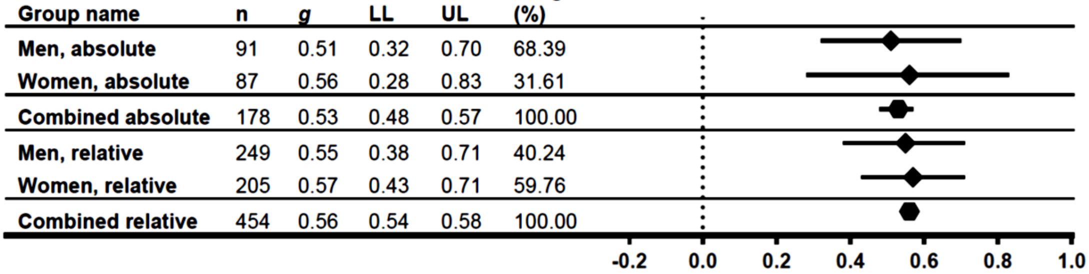

Supplementary online resource 8

a) Forest plot of pooled baseline absolute VO_{2max}, males versus females, raw mean difference

Study or Subgroup	Males Mean	Males SD	Males Total	Females Mean	Females SD	Females Total	Weight	Mean Difference IV, Random, 95% CI
Bagley 2016	3.49	0.64	24	1.99	0.454	17	14.1%	1.50 [1.17, 1.83]
Bostad 2021	3.41	0.79	6	2.15	0.42	9	7.6%	1.26 [0.57, 1.95]
Gillen 2014	3	0.5	7	2	0.2	7	12.7%	1.00 [0.60, 1.40]
Marterer 2020	4.178	0.902	9	2.327	0.526	11	7.9%	1.85 [1.18, 2.52]
Metcalfe 2016	3.01	0.57	17	2.08	0.29	18	14.8%	0.93 [0.63, 1.23]
Molina Hidalgo 2020	2.910329	0.499357	17	2.19207	0.22372	14	15.6%	0.72 [0.45, 0.98]
Søgaard 2018	2.595	0.454	11	1.873	0.15256	11	15.2%	0.72 [0.44, 1.01]
Weber 2002	3.58	0.503	7	2.55	0.29	7	12.0%	1.03 [0.60, 1.46]
**Total (95% CI)**			**98**			**94**	**100.0%**	**1.06 [0.82, 1.31]**

Heterogeneity: Tau² = 0.08; Chi² = 23.58, df = 7 (P = 0.001); I² = 70%
Test for overall effect: Z = 8.50 (P < 0.00001)

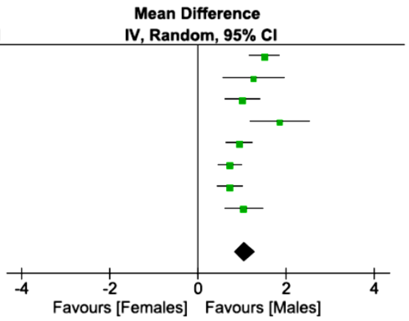

b) Forest plot of pooled baseline relative VO_{2max}, males versus females, raw mean difference

Study or Subgroup	Males Mean	Males SD	Males Total	Females Mean	Females SD	Females Total	Weight	Mean Difference IV, Random, 95% CI
Astorino 2011	45.6	4	11	41.1	6.1	9	6.3%	4.50 [-0.13, 9.13]
Bagley 2016	42.91	9.01	24	33.56	9.442	17	5.1%	9.35 [3.59, 15.11]
Gillen 2014	30.88	4.21	7	27.48	3.8	7	6.8%	3.40 [-0.80, 7.60]
Hiam 2021	47.9	8.1	74	43.1	10.1	22	6.3%	4.80 [0.19, 9.41]
Hirsch 2021	29.98	4.65	9	26.25	7.19	10	5.5%	3.73 [-1.66, 9.12]
Lepretre 2009	27	5.1	16	18.6	3.6	19	8.3%	8.40 [5.42, 11.38]
Lui 2021	56.8	7	8	42.5	2.9	8	5.6%	14.30 [9.05, 19.55]
Marterer 2020	52.3	6.2	9	39.5	4.8	11	5.9%	12.80 [7.85, 17.75]
Metcalfe 2012	36.3	5.82	7	32.5	4.24	8	5.6%	3.80 [-1.42, 9.02]
Metcalfe 2016	38.3	9.1	17	31.7	4.6	18	6.1%	6.60 [1.78, 11.42]
Molina Hidalgo 2020	37.471	5.404	17	36	4.47	14	7.7%	1.47 [-2.00, 4.95]
Mucci 2004	48.4	5	12	43.7	3.3	10	7.7%	4.70 [1.21, 8.19]
Sawashita 2009	21.5	5.144	6	19.9	2.47	17	6.7%	1.60 [-2.68, 5.88]
Scalzo 2014	43.48	6.501	11	39.56	6.99	10	5.1%	3.92 [-1.87, 9.71]
Schubert 2017a	37.225	8.334	10	28.06	5.56	14	4.9%	9.17 [3.24, 15.09]
Søgaard 2018	27.4	6.633	11	23.1	3.32	11	6.6%	4.30 [-0.08, 8.68]
**Total (95% CI)**			**249**			**205**	**100.0%**	**5.88 [4.09, 7.67]**

Heterogeneity: Tau² = 7.68; Chi² = 37.23, df = 15 (P = 0.001); I² = 60%
Test for overall effect: Z = 6.45 (P < 0.00001)

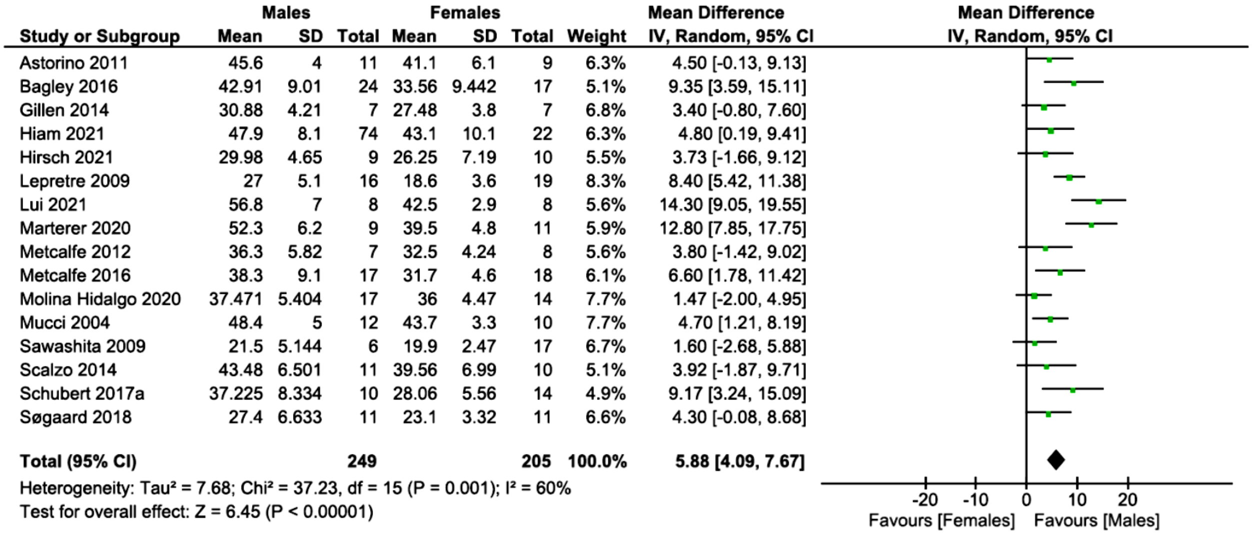

c) Forest plot of pooled pre-post absolute VO_{2max}, sub-grouped as males and females, raw mean difference

Study or Subgroup	Post Mean	Post SD	Post Total	Pre Mean	Pre SD	Pre Total	Weight	Mean Difference IV, Random, 95% CI
**2.2.1 Males**								
Bagley 2016	3.68	0.73	12	3.49	0.64	12	4.4%	0.19 [-0.36, 0.74]
Bostad 2021	4.2	0.75	3	3.41	0.79	3	0.9%	0.79 [-0.44, 2.02]
Marterer 2020	4.594	0.782	4	4.178	0.902	5	1.1%	0.42 [-0.69, 1.52]
Metcalfe 2016	3.28	0.53	8	3.01	0.57	9	4.8%	0.27 [-0.25, 0.79]
Molina Hidalgo 2020	3.39275	0.49414	9	2.910329	0.499357	8	5.9%	0.48 [0.01, 0.96]
Søgaard 2018	2.796	0.58041	6	2.595	0.45438	5	3.5%	0.20 [-0.41, 0.81]
Weber 2002	3.85	0.4498	3	3.58	0.5027	4	2.6%	0.27 [-0.44, 0.98]
**Subtotal (95% CI)**			**45**			**46**	**23.2%**	**0.32 [0.09, 0.56]**
Heterogeneity: Tau² = 0.00; Chi² = 1.45, df = 6 (P = 0.96); I² = 0%								
Test for overall effect: Z = 2.67 (P = 0.008)								
**2.2.2 Females**								
Bagley 2016	2.37	0.49	8	1.99	0.454	9	6.5%	0.38 [-0.07, 0.83]
Bostad 2021	2.49	0.57	5	2.15	0.42	4	3.1%	0.34 [-0.31, 0.99]
Marterer 2020	2.512	0.495	5	2.327	0.526	6	3.6%	0.19 [-0.42, 0.79]
Metcalfe 2016	2.29	0.37	9	2.08	0.29	9	14.0%	0.21 [-0.10, 0.52]
Molina Hidalgo 2020	2.4939	0.23733	7	2.19207	0.22372	7	22.6%	0.30 [0.06, 0.54]
Søgaard 2018	1.926	0.27196	5	1.873	0.15256	6	18.4%	0.05 [-0.21, 0.32]
Weber 2002	2.62	0.2117	4	2.55	0.291	3	8.7%	0.07 [-0.32, 0.46]
**Subtotal (95% CI)**			**43**			**44**	**76.8%**	**0.20 [0.07, 0.33]**
Heterogeneity: Tau² = 0.00; Chi² = 3.07, df = 6 (P = 0.80); I² = 0%								
Test for overall effect: Z = 3.02 (P = 0.002)								
**Total (95% CI)**			**88**			**90**	**100.0%**	**0.23 [0.12, 0.35]**
Heterogeneity: Tau² = 0.00; Chi² = 5.30, df = 13 (P = 0.97); I² = 0%								
Test for overall effect: Z = 3.94 (P < 0.0001)								
Test for subgroup differences: Chi² = 0.78, df = 1 (P = 0.38), I² = 0%								

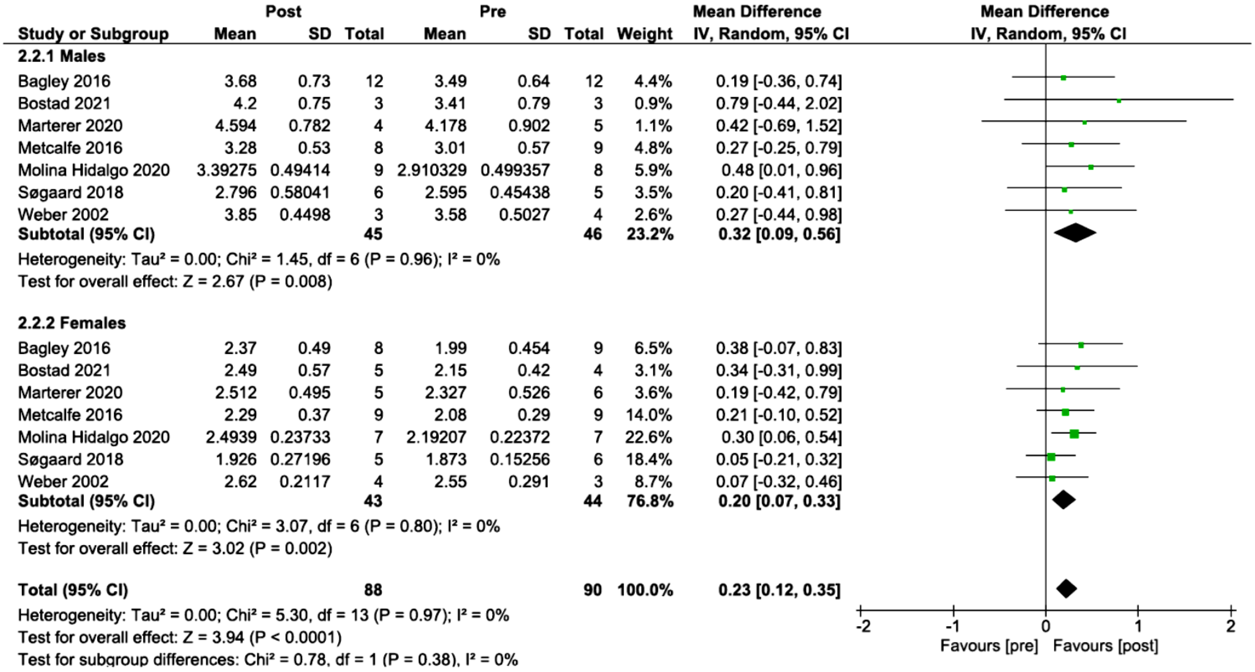

d) Forest plot of pooled pre-post relative VO_{2max}, sub-grouped as males and females, raw mean difference

Study or Subgroup	Post Mean	Post SD	Post Total	Pre Mean	Pre SD	Pre Total	Weight	Mean Difference IV, Random, 95% CI
**2.1.1 Males**								
Astorino 2011	48.3	5.2	5	45.6	4	6	3.2%	2.70 [-2.87, 8.27]
Bagley 2016	45.49	7.25	12	42.91	9.01	12	2.4%	2.58 [-3.96, 9.12]
Gillen 2014	34.43	4.1	3	30.88	4.1	4	2.7%	3.55 [-2.59, 9.69]
Hiam 2021	48.9	8.2	37	47.9	8.2	37	7.2%	1.00 [-2.74, 4.74]
Hirsch 2021	35.83	4.74	5	29.98	4.21	4	2.9%	5.85 [-0.01, 11.71]
Lepretre 2009	29.9	4.5	8	27	5.1	8	4.5%	2.90 [-1.81, 7.61]
Lui 2021	63.6	4.7	4	56.8	7	4	1.5%	6.80 [-1.46, 15.06]
Marterer 2020	58.1	4.7	5	52.3	6.2	4	1.9%	5.80 [-1.54, 13.14]
Metcalfe 2012	41.6	3.97	4	36.3	5.82	3	1.7%	5.30 [-2.35, 12.95]
Metcalfe 2016	41.4	8.9	9	38.3	9.1	8	1.4%	3.10 [-5.48, 11.68]
Molina Hidalgo 2020	43.65	5.4	8	37.471	5.404	9	3.8%	6.18 [1.03, 11.32]
Mucci 2004	53.1	5.1	6	48.4	5	6	3.1%	4.70 [-1.01, 10.41]
Sawashita 2009	24.7	9.31	3	21.5	5.144	3	0.7%	3.20 [-8.84, 15.24]
Scalzo 2014	44.95	7.16	6	43.48	6.501	5	1.5%	1.47 [-6.61, 9.55]
Schubert 2017a	38.89	8.72	5	37.225	8.334	5	0.9%	1.66 [-8.91, 12.24]
Søgaard 2018	29.5	6.63	5	27.4	6.633	6	1.6%	2.10 [-5.77, 9.97]
**Subtotal (95% CI)**			**125**			**124**	**41.0%**	**3.50 [1.93, 5.06]**
Heterogeneity: Tau² = 0.00; Chi² = 5.46, df = 15 (P = 0.99); I² = 0%								
Test for overall effect: Z = 4.38 (P < 0.0001)								
**2.1.2 Females**								
Astorino 2011	43.9	5.7	4	41.1	6.1	5	1.7%	2.80 [-4.93, 10.53]
Bagley 2016	39.84	10.06	9	33.56	9.442	8	1.2%	6.28 [-2.99, 15.55]
Gillen 2014	30.78	4.51	4	27.48	3.8	3	2.6%	3.30 [-2.87, 9.47]
Hiam 2021	44.1	9.4	11	43.1	10.1	11	1.5%	1.00 [-7.15, 9.15]
Hirsch 2021	30.47	8.46	5	26.25	7.19	5	1.1%	4.22 [-5.51, 13.95]
Lepretre 2009	21.1	3.7	9	18.6	3.6	10	9.3%	2.50 [-0.79, 5.79]
Lui 2021	46.2	3	4	42.5	2.9	4	6.0%	3.70 [-0.39, 7.79]
Marterer 2020	43.1	5.8	6	39.5	4.8	5	2.6%	3.60 [-2.66, 9.86]
Metcalfe 2012	36.4	3.68	4	32.5	4.24	4	3.3%	3.90 [-1.60, 9.40]
Metcalfe 2016	34.7	5.2	9	31.7	4.6	9	4.9%	3.00 [-1.54, 7.54]
Molina Hidalgo 2020	40.8	5.13	7	36	4.47	7	4.0%	4.80 [-0.24, 9.84]
Mucci 2004	46.7	5	5	43.7	3.3	5	3.7%	3.00 [-2.25, 8.25]
Sawashita 2009	26.9	5.77	9	19.9	2.47	8	5.9%	7.00 [2.86, 11.14]
Scalzo 2014	42.55	3.89	5	39.56	6.99	5	2.0%	2.99 [-4.02, 10.00]
Schubert 2017a	29.91	5.93	7	28.06	5.56	7	2.8%	1.85 [-4.17, 7.87]
Søgaard 2018	23.9	3.32	6	23.1	3.32	5	6.5%	0.80 [-3.14, 4.74]
**Subtotal (95% CI)**			**104**			**101**	**59.0%**	**3.34 [2.03, 4.64]**
Heterogeneity: Tau² = 0.00; Chi² = 6.28, df = 15 (P = 0.97); I² = 0%								
Test for overall effect: Z = 5.00 (P < 0.00001)								
**Total (95% CI)**			**229**			**225**	**100.0%**	**3.40 [2.40, 4.41]**
Heterogeneity: Tau² = 0.00; Chi² = 11.77, df = 31 (P = 1.00); I² = 0%								
Test for overall effect: Z = 6.65 (P < 0.00001)								
Test for subgroup differences: Chi² = 0.02, df = 1 (P = 0.88), I² = 0%								

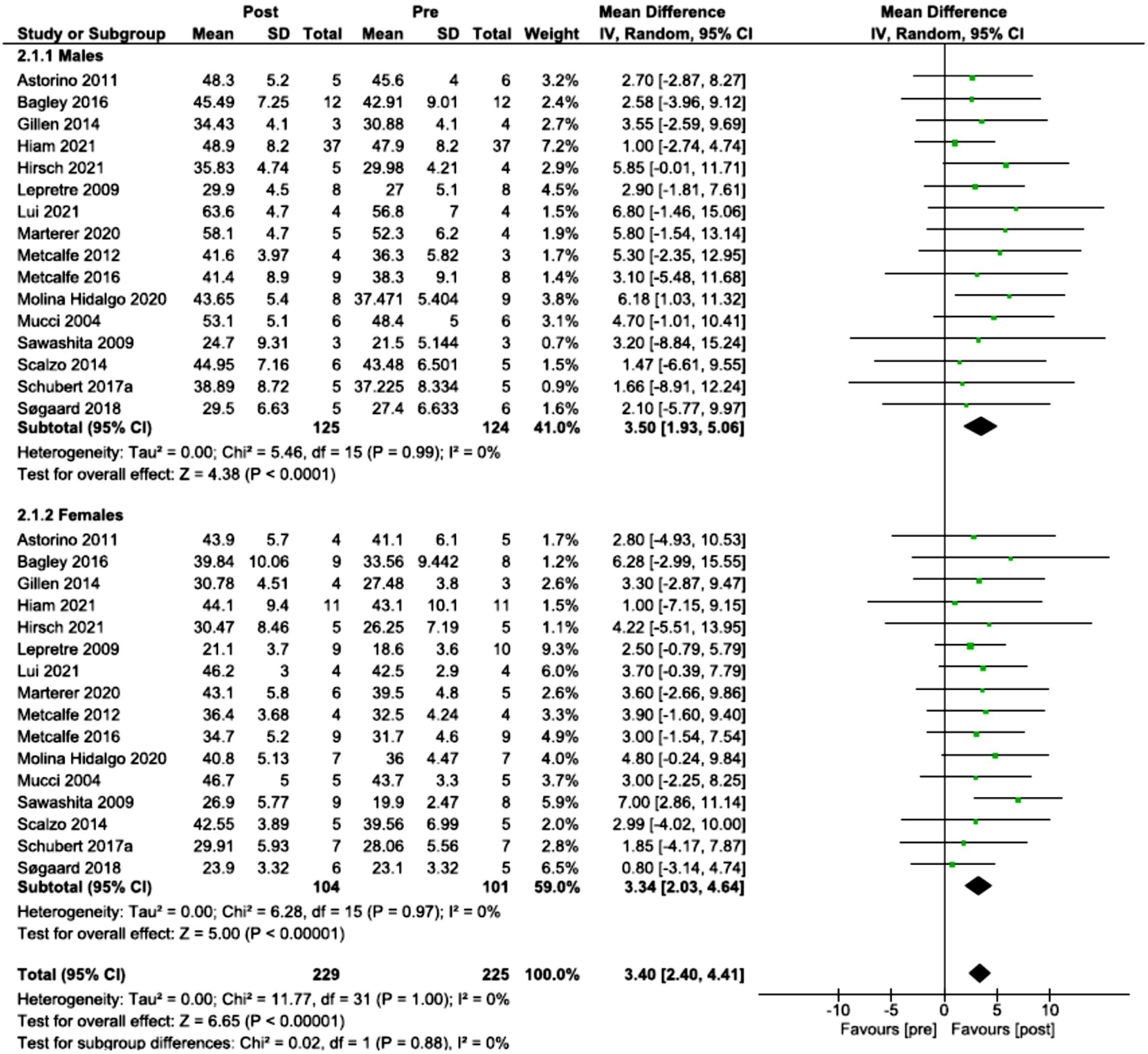

Supplement: Supplementary file 2 — Supplementary file2 (PDF 1577 KB) [file 40279_2023_1914_MOESM2_ESM.pdf]
